# The impact of exercise on skeletal muscle proteome of prediabetic subjects analyzed with data independent mass spectrometry

**DOI:** 10.1038/s41598-025-13942-z

**Published:** 2025-08-07

**Authors:** Anna Czajkowska, Łukasz Szczerbiński, Marcin Czajkowski, Anna Citko-Rojewska, Paulina Konopka, Agnieszka Blachnio-Zabielska, Adam Krętowski, Piotr Zabielski

**Affiliations:** 1 Department of Medical Biology, Medical University of Bialystok, Bialystok, Poland; 2 Department of Endocrinology, Diabetology and Internal Medicine, Medical University of Bialystok, Bialystok, Poland; 3 Faculty of Computer Science, Bialystok University of Technology, Bialystok, Poland; 4 Regional Digital Medicine Centre, Medical University of Bialystok, Bialystok, Poland; 5 Department of Hygiene, Epidemiology and Metabolic Disorders, Medical University of Bialystok, Bialystok, Poland; 6 Clinical Research Centre, Medical University of Bialystok, Bialystok, Poland; 7 Programs in Metabolism and Medical & Population Genetics, Broad Institute of MIT and Harvard, Cambridge, MA USA

**Keywords:** Proteomics, Endocrine system and metabolic diseases, Hormones, Type 2 diabetes, Metabolic syndrome, Obesity, Pre-diabetes, Skeletal muscle, Predictive markers, Type 2 diabetes, Mass spectrometry, Medical and clinical diagnostics, Biochemical networks, Proteomics

## Abstract

Physical exercise of even a moderate intensity is beneficial in both the prevention of prediabetes and management of Type 2 diabetes mellitus, as skeletal muscle is a primary tissue responsible for glucose uptake. Exercise-evoked proteomic alterations in muscle of subjects with prediabetes are of great importance for the study of relationships between insulin resistance and exercise. Although data-dependent (DDA) proteomic analysis is a cornerstone of deep proteome profiling employed in the elucidation of skeletal muscle biology, data-independent (DIA) approaches gain popularity in the studies focused on data reproducibility and throughput. We compared various ion-chromatogram libraries assembled with the use of off-line high-pH fractionation (HpH), gas-phase fractionation (GPF) and libraryless DirectDIA in LC/MS/HRMS DIA proteomic analysis of muscle from normoglycemic (NGT) and prediabetic (IGT) subjects after 3 months of supervised, mixed-mode exercise. In our hands, GPF-fractionated, hybrid DDA/DIA libraries yielded the best overall balance between the speed of preparation, data collection and protein identification among tested approaches. Analysis revealed, that despite 3-month exercise intervention skeletal muscle from IGT subjects displayed significant alterations in pathways and molecules relevant to muscle contraction, extracellular matrix composition and protein synthesis as compared to NGT counterparts. In conclusion, our study underlines the importance of the ion library assembly in the DIA analysis of clinical samples and confirms at molecular level changes connected with deficiency of muscle function in the prediabetic state.

## Introduction

Type 2 diabetes mellitus (T2DM) and its comorbidities such as cardiovascular-related disorders, hyperlipidemia, obesity, nephropathy, neuropathy and decrease in cognitive function and dementia become a major health-related burden worldwide^1,2^. Progression towards T2DM is connected with increasing insulin resistance of major insulin-sensitive tissues (muscle, adipose tissue, liver) with subsequent induction of pre-diabetic state. This usually asymptomatic, obscure metabolic abnormality can manifest itself as isolated impaired fasting glucose (IFG – fasting blood glucose between 100mg/dl and 125mg/dl) or impaired glucose tolerance (IGT – 2 h blood glucose after 75g oral glucose challenge between 140mg/dl and 199mg/dl) and elevated blood insulin^3^. As prediabetes is often overlooked, it progresses toward symptomatic T2DM after β-cell failure, when pancreatic islets are unable to compensate for increasing systemic insulin demands. This crucial watershed event marks an irreversible stage of T2DM pathogenesis^4,5^. Currently, due to development of various pharmacotherapies (SGLT2 inhibitors, DPP4 inhibitors, GLP1 analogs and receptor agonists, insulin sensitizers and long-acting insulin analogs) case-tailored T2DM management is increasingly successful, leading to both a decrease in hyperglycemia and T2DM comorbidities^6,7^. Contrary to fully developed T2DM, β-cell dysfunction and systemic insulin resistance in prediabetes is reversible and can be successfully managed by approaches such as bariatric surgery, pharmacotherapy, lifestyle changes, physical exercise and their combinations^8–11^. From the above, physical exercise appears as an easily accessible, universal remedy in both the prediabetes prevention, augmentation of its reversal or inhibition of its progression towards T2DM^10,12,13^. Moreover, physical exercise has shown beneficial effects in the prevention of other metabolic-syndrome connected comorbidities such as cognitive impairment and dementia^14–16^.

Metabolic health of muscle tissue is of great importance for both the physical fitness and whole-body energy metabolism. As a major tissue in both insulin-stimulated and insulin-independent (exercise-evoked) uptake and metabolism of plasma glucose, its role in prediabetes prevention and its reversal cannot be overlooked. Prediabetes was shown to induce detrimental effects in both the metabolic and contractile muscle function^17–20^. Crucially, exercise in different forms and modalities was shown to improve both metabolic and functional abnormalities observed in prediabetes^21,22^. Taking all of the above into account, analysis of the impact of exercise on the proteome of prediabetic skeletal muscle is crucial for the elucidation of its beneficial effects. Moreover, the subject of equal importance is the identification of persistent negative effects of prediabetes, which are not fully corrected by the physical activity. Proteomic analysis of skeletal muscle significantly improved our understanding of muscle physiology and pathology. Bottom-up data-dependent proteomic analysis (DDA—where individual peptide precursor ions are sequentially selected for fragmentation based on their intensity in survey spectrum) was extensively used for deep muscular proteome profiling, yielding novel data on fiber type specific skeletal muscle adaptations to exercise training^23^, convergent effects of acute exercise or insulin stimulation on muscular phosphoproteome^24^ or muscular protein–protein interactome in healthy and obese T2DM subjects^25^. Versatility of DDA approach arises from proven reliability and accessible implementation, both at the level of equipment and bioinformatic resources. Moreover, the DDA ability to reliably interpret MS data acquired from fractionation-based experiments (both at the protein or peptide levels) overcomes the detrimental impact of high-abundance proteins on overall proteome coverage. This can be an issue in skeletal muscle bottom-up proteomic analysis, as it is mainly composed of chains of biological polymers involved in muscle contraction, such as myosin, actin, tropomyosin and titin chains^26,27^, which can subsequently mask low-abundant proteins^28,29^. Finally, DDA is preferred method of choice in the multiplex assays, performed with the use of TMT or iTRAQ mass tags, which improves both the identification rate and reproducibility of the measurement^24^. Nevertheless, the expansion of MS-based proteomic analysis beyond basic science research towards routine assays was a driving force in development of methods focused on increased reproducibility and speed of acquisition—the features as crucial as extensive proteome coverage characteristic to DDA-based profiling. Data-independent analysis (DIA – where all the peptide precursor ions are fragmented in the wider isolation window) gains in popularity as it offers increased reproducibility and throughput^30–33^. Recently, it was also employed together with PASEF ion mobility to study personalized signatures of insulin resistance in type 2 diabetic subjects^34^.

Robust methods for proteomic analysis of large cohorts of clinical skeletal muscle samples need to address the specificity of this tissue at both the LC/MS/MS level and subsequent bioinformatic analysis. At the HPLC peptide separation level, it requires the use of traps and columns resistant to clogging by long biological polymers, flow-reversible for easy cleanup and restoration of its initial performance, with robust retention time stability after hundreds of samples^35,36^. Ideally, with low operating pressure,long separation length and adequate resolution to address the excess of peptides from contractile proteins characteristic to striated skeletal muscle. At the MS level, it dictates the use of data-independent acquisition (DIA), ideally supplemented with retention time calibration peptides, which is better suited for clinical applications due to its reproducibility and non-stochastic nature of identification, unlike data-dependent acquisition (DDA)^33,37^. Finally, employment of DIA requires assembly of appropriate ion-chromatogram libraries, or selection of libraryless, pure bioinformatic identification and quantitation pipeline^38–40^.

To address above challenges, we constructed a pipeline tailored for proteomic analysis of clinical skeletal muscle biopsies. We employed μPAC semiconductor-technology chromatography columns and traps, retention-time calibration peptides and staggered-windows DIA analysis to address reproducibility issues inherent to challenging sample matrices. As employment of different approaches to ion-chromatogram library creation can lead to distinct biological findings, we compared various ion-chromatogram libraries based on off-line high-pH fractionation (HpH), gas-phase fractionation (GPF) and libraryless DirectDIA approach. Finally, we performed proteomic analysis of muscle from normoglycemic (NGT) and prediabetic (IGT) subjects after 3 months of supervised, mixed-mode exercise.

## Materials and methods

### Study participants and sample collection

The muscle biopsy samples were collected from participants of the “Bialystok Exercise Study in Diabetes (BESD)”, conducted by the Department of Endocrinology, Diabetology and Internal Medicine and Clinical Research Centre of the Medical University of Bialystok^22,41^. The study and its procedures were approved by the Ethics Committee of the Medical University of Bialystok (No. R-I-002/469/2014). All methods were carried out in accordance with relevant guidelines and regulations, including the Declaration of Helsinki. Written informed consent was obtained from each participant prior to participation and sample collection. Briefly, the study cohort involved physically inactive men with various degrees of dysglycemia, with a BMI of 25–35 kg/m^2^ and leading a sedentary lifestyle. All participants underwent three-month exercise program that included supervised training sessions at a local fitness center. Studied patients participated in a 3-month exercise intervention consisting of mixed training, aerobic and strength exercises, performed for 85 min 3 times a week, totaling in 36 sessions over 12 weeks^22,41^. The routine started with a 15-min warm-up, followed by 40 min of strength exercise of major muscle groups (60–75% of single repetition maximum, adjusted weekly) and 30 min of moderate-intensity endurance exercise (60–70% of individual VO_2_max), all supervised by an exercise technician. The myWellness system (Technogym, Cesena, Italy) recorded each session for consistency. Dietary supervision aimed to eliminate diet-related variables. For the assessment of proposed methodology, we selected only the post-exercise bioptates from non-diabetic, normoglycemic, with normal glucose tolerance (NGT group; n = 13, fasting plasma glucose (FPG) < 100 mg/dL and 2-h glucose during OGTT (2h-OGTT-GLU) < 140 mg/dL) and prediabetic, impaired glucose tolerance group (IGT group; n = 11; FPG < 100 mg/dL, 2h-OGTT-GLU 140–199 mg/dL). Analysis was performed on 2 independent bioptates from each participant, yielding a total of 26 NGT and 22 IGT samples (total of 48 individual samples). Post-exercise anthropometric characteristics of NGT and IGT groups are presented in Supplement 1 (Table S1). The vastus lateralis muscle (VL) biopsies were obtained from fasted participants 48 h after the last exercise session with the use of a percutaneous suction needle^42^. Excess blood, connective tissue and fat was removed after visual inspection immediately after collection. All samples were snap-frozen and stored in LN_2_ until further analysis.

### Experimental design

**Fig. 1:**
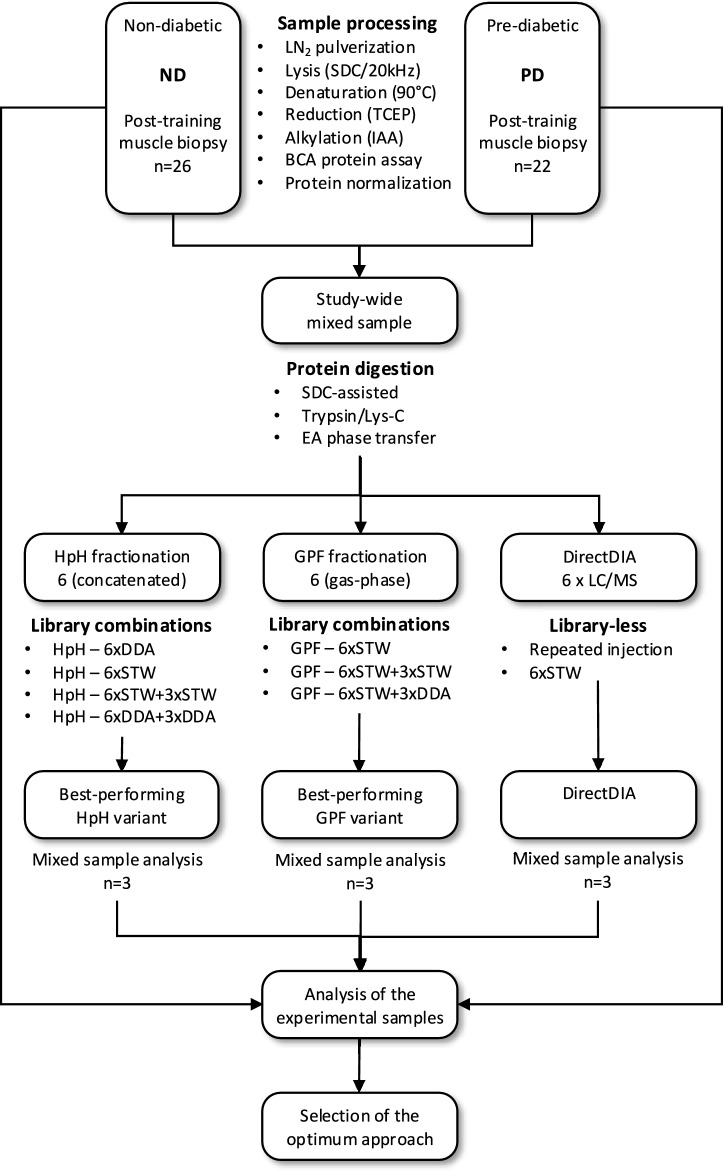
Schematics of the study design. Proteins from post-training, vastus lateralis muscle LN_2_ pulverizates were extracted with SDC-assisted lysis and sonication. Approx. 20 µg of protein from each biopsy was combined into study-wide mixed sample. This sample – after digestion – was used to create ion-chromatogram libraries through High-pH fractionation with fraction concatenation (HpH, 6 final fractions), gas-phase fractionation approach (GPF, 6 fractions) or repetitive injections (libraryless DirectDIA approach, 6 repetitive injections). Assessed library combinations included data collected in DDA mode, STW mode (staggered windows DIA), or both. Subsequently, to assess performance of approaches in protein identification and quantitation, three best-performing library combinations were used to analyze data from mixed muscle samples collected in STW mode. Finally, the best-performing libraries were utilized for the IGT vs. NGT differential protein expression analysis across the entire set of experimental samples.

Experimental design of the study is presented in Fig. 1.

### Sample preparation

Protein extraction and digestion was performed with the use of sodium deoxycholate-assisted sample preparation with phase transfer SDC extraction and lipid depletion technique according to Leon et al.^43^. Briefly, approx. 25-30mg of lateral thigh muscle biopsy was pulverized in LN_2_, suspended in tissue lysis buffer (1:10 w/v; 50mM ammonium bicarbonate (ABC), 5% sodium deoxycholate (SDC), 5mM TCEP) and sonicated for 2 × 30 s. on ice at 50% power (Sonics&Materials VCX-130 Ultrasonic Processor). Subsequently, samples were denaturated by heating for 30 min. at 60°C, alkylated with iodoacetamide (IAA) for 30 min. at room temperature (IAA, 1M in ABC buffer, 15mM final concentration) and centrifuged for 5min. at 14000g to sediment tissue debris. Protein concentration was measured with BCA assay (reducing-agent compatible, Thermo Scientific) and protein content was normalized to 2.5 μg/μl with 5% SDC in 50mM ABC buffer. Digestion-ready samples were stored in -80°C until further analysis. For sample digestion, 10μl of sample equivalent to 25μg of protein, was diluted to 90μl with 50mM ABC. Trypsin/LysC mix (2.5μl from 0.4μg/μl stock in 50mM acetic acid) was added at a ratio of 1:25 enzyme/protein (1:50 ratio for each of the enzymes). Samples were incubated at 37°C for 12h with shaking (600 rpm) in Thermoblock, and subsequently cooled and kept at 4°C until next working day. After addition of TFA to 0.5% concentration and brief vortexing, precipitated SDC and lipids were extracted 3 times with 100μl of ethyl acetate. Each time samples were vortexed and centrifuged for 5 min at 14000g. Residual ethyl acetate was evaporated by heating the open tubes at 60°C for 30 min with gentle shaking. Subsequently, 10 injection equivalents of iRT peptides (Biognosys) were added to each sample. Additionally, approx. 20μg of protein was taken from each homogenized biopsy to create study-wide mixed sample. After protein digestion, this mixed sample was used for offline high-pH fractionation (HpH), gas-phase fractionation (GPF) and for repetitive injections performed at the library selection stage. All injection-ready samples were stored in -80°C until further analysis.

### LC/MS/MS analysis

The same optimized chromatographic conditions were used for both data-dependent (DDA) and data-independent (DIA) analysis. Detailed description and results of optimization of chromatographic gradient, column flow and sample loading for µPAC pillar array trap and column are presented in Supplement 1 (Table S2–S4). All optimization steps were performed on HeLa digest standard (Thermo Scientific) run in triplicate in DDA mode.

LC/MS/MS analysis was performed on Q-Exactive MS equipped with IonFlex II source and silica glass emitter (10μm or 20μm I.D. PicoTip, flow-rate dependent; NewObjective) working at 1.75kV in + ESI mode. Thermo Scientific 3500RSLC nanoLC configured for trap-elute was used in all chromatographic separations. For standard runs, samples were loaded at 750ng of peptides onto µPAC nano pillar array trap at 10μl/min (2% ACN, 0.2% TFA) and resolved on 50cm µPAC pillar array column (channel length 500mm, channel width 315μm, pillar height 18μm, pillar diameter 5μm, interpillar distance 2.5μm) at 300nl/min using 115min multi step reverse-phase binary gradient (A – 0.2% FA in H_2_O, LC/MS; B – 0.2% FA in 90% acetonitrile, LC/MS; 5%B-4min, 30%B-71min, 45%B-94min, 90%B-95min, 90%B-103min, 5%B-104min, 5%B-115min). Total sample run time excluding autosampler draw equaled 120min. Daily routine started with mass calibration, nanoLC solvent purge and column equilibration. Each batch of 11 samples was preceded by iRT-spiked QC sample (300ng of HeLa digest standard, Thermo Scientific) analyzed with 55 min gradient and DDA acquisition. Study-wide analysis of sample-spiked iRT peptide retention time variation, FWHM and mass accuracy and total ion chromatograms of all experimental samples and respective mass accuracy at MS and MS/MS level is presented in Supplement 1 (Figure S1 and S2). The effects of signal normalization at the MS/MS level for all the experimental samples analyzed using HpH/DDA-H, GPF/STW-H, and DirectDIA methods are presented in Supplement 1 (Figure S4).

### Data-dependent (DDA) acquisition

DDA analysis of eluting peptides for both the optimization of chromatographic conditions and library generation was performed using Top 20 method with following settings: MS1 resolution of 70k, ACG target 3e^6^, max. IT 50ms, scan range 385-1015m/z; MS2 resolution 17k, ACG target 2e^5^, max. IT 55ms, isolation 2m/z with 0.4mz offset to include most of isotopologues in case of multiply-charged peptides. Top 20 peptides (excluding isotopes) were selected for fragmentation with normalized collision energy (NCE) lowered to 27, if following criteria were fulfilled: charged state 2–5, preferred peptide match, min. ACG 6e^3^ and intensity threshold 1.1e^5^, with dynamic exclusion set at 15s.

### Data-independent (DIA) acquisition and gas-phase fractionation (GPF)

DIA method for library generation and sample analysis (referred as 24m/z STW in the manuscript) was designed on the basis of staggered windows approach by Searle, Pino and Amodei^38,44–46^. Method consisted of 52 staggered, 24m/z wide windows (12m/z overlap), covering 400–1000 m/z (referred as 24SW method) with MS1 scan covering 385-1015m/z performed after 26 windows (Supplement 1, Figure S3; Supplement 2, Table S1). Mass range was selected on the basis of distribution of peptide m/z values from DDA run of mixed muscle sample. Window edges were placed in peptide forbidden zones to account for non-rectangular characteristics of Q-Exactive quadrupole mass isolation. Window placement was designed with Skyline. MS1 resolution was set at 35k, ACG at 1e^6^ and max. IT of 60ms. Windowed MS2 was performed at 17k resolution, with ACG at 1e^6^, max. IT of 60ms, MSX count of 1 with isochronous IT set to ON and 27 NCE at default charge state of 3.

For gas-phase fractionation (GPF) with staggered windows approach (4 m/z GPF-STW), 750ng of peptides was resolved for each of the 6 gas-phase fractions. Gas-phase fractionation consisted of 6 overlapping segments 102m/z wide (2m/z overlap) jointly covering 400–1000 m/z mass range (Supplement 2, Table S2). Each gas-phase fraction consisted of 52 staggered, 4 m/z wide windows (2m/z overlap), with MS1 scan covering respective mas range performed after 26 windows and utilized identical MS1 and MS2 settings as DIA method described above. Results of GPF with staggered windows presented in Supplement 1 (Figure S3).

### Off-line high-pH peptide fractionation with fraction concatenation

For HpH fractionation with fraction concatenation (HpH), post-digestion peptides from mixed sample were evaporated to dryness using Labconco Vacuum Concentrator at 45°C under 0.05atm residual pressure. Subsequently, peptides were dissolved at 5μg/μl in 2% ACN in 100mM NH_4_OH with 0.001% Zwittergent 3–16 through vortexing and brief sonication. Up to 100μg (20μl) of peptides were loaded onto C18 Waters X-Bridge Peptide BEH Column (300Å, 3.5 µm, 1 mm X 150 mm) and resolved using Dionex 3500RSLC. Peptides were eluted at a flow rate of 100μl/min using a 45 min, reverse-phase binary gradient (A -2% ACN in 10mM NH_4_OH, B – 90% ACN in 10mM NH_4_OH; gradient 3.3%B for 2.5min, 25%B at 32.5min, 40%B at 35min, 90%B at 35.5min for 3.5min, 3.3%B at 42.5min for 2.5min) under UV monitoring at 214nm (peptide bond) and 280nm (tyrosine, tryptophan). Peptide fractions eluting for up to 42 min were collected every 140 s into 96 well 500µl LoBind plate (Eppendorf). Resulting 18 fractions were pooled to final 6 using concatenation scheme to enhance orthogonality of basic (high-pH) and acidic (low-pH) separations and equalize concentration and distribution of peptides. Combined peptide fractions were evaporated under vacuum and resuspended at 250ng/μl in sample loading buffer (0.5% TFA, 2% ACN, 0.001% Zwittergent). Spike of iRT peptides was added to each of concatenated samples. Subsequently, 1μg of peptides from each fraction was analyzed by both the DDA and DIA methods and resulting data was used to generate appropriate library combination. Results of HpH fractionation are presented in Supplement 1 (Figure S5).

MS cycle time of 3180ms was matched across all the methods and chromatographic runs to give approx. 8 points per peak at FWHM (Supplement 1; Table S6). For all DDA, STW and GPF-STW methods, polysiloxane at 445.12003 m/z was used as lock mass for real-time calibration.

### Ion-chromatogram libraries

To select optimal analysis approach, several versions of libraries were compiled. Each included combinations of appropriate fractions (GPF, HPH, collected in DDA and STW mode) and mixed sample runs (collected in STW mode). Spectronaut version 15.4.210913.50606-Rubin^47^, was used to search raw files with the build-in Pulsar engine against Homo sapiens Uniprot reference proteome FASTA file (UP000005640_9606, one sequence per protein). Pulsar standard settings were used, with following exceptions: digestion by Trypsin/LysC with P, carbamidometylation of C as fixed, acetylation of N-terminal, methylation of K or R, deamidation of Q or N, oxidation of M as variable modifications. Peptide length between 7 and 35 amino acids, max 5 variable modifications and max 2 missed cleavages per peptide. Spectra collected with staggered windows method were demultiplexed by build-in algorithm^46^. Dynamic mass calibration was used to correct both MS1 and MS2 spectra. Library was constrained to include from 3 to 6 of most intense b or y type fragments (300mz – 1800m/z range) per peptide of at least 3AA in length. Universal 1% FDR cutoff was used at the level of precursors; peptides and proteins. Retention times and RT window widths were calibrated using deep learning-assisted iRT regression included in Spectronaut. Results of library generation step are presented in Supplement 1 (Table S5).

Subsequently, mixed sample runs collected with standard 24m/z STW method (n = 3) were searched with different versions of libraries to select best-performing combinations. The results are presented in Supplement 1 (Table S6).

### Data analysis

Biognosys Spectronaut was used to extract ion chromatograms from MS1 and MS2 data based on both the maximum ion peak intensity using dynamic mass tolerance, dynamic iRT-corrected retention time window and global, MS2-level signal normalization (Supplement 1 Figure S3). Subsequently, data was searched with selected experiment-specific ion libraries. Q-value of 0.01 for both the precursors and peptides (corresponding to 1% FDR) was at the experiment level with a target-decoy approach using mutated sequences. For proteins Q-value as set at < 0.05 (5% FDR) and < 0.01 (1% FDR) at run-vise and experiment-vise levels, respectively. Quantification was performed at MS2 level, with Spectronaut bulid-in MaxLFQ algorithm. Within-sample peptide quantity was calculated as a mean of the peak areas of top 3 MS2 fragment ions, whereas protein quantity was expressed as a mean of top 3 peptides. For NGT and IGT group comparison, only the proteins present in at least 50% of the samples (Q-value percentile 0.5), with 2 unique peptides, presenting at least 50% expression difference (-0.585 ≥ log_2_(fold change) ≥ 0.585) and FDR-adjusted p-value < 0.05 (Q-value < 0.05, -log_10_(p-value) > 1.13) were regarded as significant^47^. For molecular pathway and molecular interaction analysis, IGT/NGT protein Log2 expression rations and Q-values were uploaded to Quiagen Ingenuity Pathway Analysis (IPA, version 2023.4 Fall Release, Qiagen, https://digitalinsights.qiagen.com/IPA)^48^. Both expression Q-value cutoffs were used to restrict dataset to only significantly affected proteins (as mentioned above). IPA Core Analysis was restricted to mammalian proteins (human, rat, mouse) present in skeletal muscle, using stringent filtering for both molecules and interactions. Z-scores for pathways, diseases and bio functions were calculated based on expression log2 ratios. Corresponding -log_10_(p-value) of overlap were calculated using right-tailed Fisher’s exact test with and FDR correction. Compare analysis function in IPA was used to correlate IPA results from IGT/NGT comparison generated with the HpH, GPF and DirectDIA approach. Results of comparison analysis at molecular pathway, disease and bio-function and individual protein level are presented in Supplement 2 (Table S3-S5). Detailed results of IPA analysis performed on samples quantified with respective library are presented in Supplement 2 (Table S6-S8). Protein functional clustering was performed with String^49^. We conducted a standard gene set analysis of significantly affected proteins, using their gene names and ranked FDR-adjusted p-values (4 ranks) expressed as -log_10_(p-value). Analysis was performed with full STRING Homo sapiens database (text mining, experiments, databases, co‑expression, neighborhood, gene fusion, co‑occurrence), confidence of supporting data was used as node connection (network edge) and minimum confidence of interaction was set at 0.500 (medium–high). Disconnected nodes were discarded. Clustering was performed using k-means and minimum number of clusters of 3. K-parameter (cluster number) was automatically addressed. Functional network enrichment was performed with FDR ≤ 1% and strength ≥ 0.75. The ranked -log_10_(p-value) significance of change in IGT/NGT protein expression was used as node halo shading in network visualization. Full results of STRING cluster analysis are presented in Supplement 2 (Table S9-S11).

## Results

### Utilization of μPAC column for LC/MS/HRMS proteomic analysis of skeletal muscle biopsy

The use of 50cm semiconductor-technology micro pillar array columns (μPAC) dictated separate gradient optimization steps, as the direct employment of gradients optimized for grain-based 50 microcapillary columns gave sub-par identification values. Best results were obtained with the use of multi-step (Table S2), 300nl/min 120min gradient (Table S3) at column load of 750ng of skeletal muscle digest (Table S4). Median CV% of retention time variation for all sample-spiked iRT peptides equaled to 26s, with FWHM of 17s (Figure S1 A and B). Pillar array column delivered good chromatographic resolution, retention time stability and reproducible detector signal (Figure S1C and D) accompanied by exceptionally low operating pressure as compared to typical, high-performance, particle-based capillary chromatography column of comparable length (Figure S1D).

### Initial comparison of methods for ion-chromatogram library generation

At the early stage of ion-chromatogram library construction, we compared total number of identifications at peptide and protein level for mixed, study-wide muscle sample. HpH fractionation with fraction concatenation (Supplement 1, Figure S6) yielded highest identification numbers, at the level of both the individual and combined fractions. HpH outperformed in this regard both GPF (Supplement 1, Figure S5) and DirectDIA (Supplement 1, Figure S7) performed on 6 constitutive samples. As the results from HpH and GPF fractionation include runs from 6 independent fractions, we compared them with the values from 6 constitutive non-fractionated sample runs analyzed with DirectDIA approach. Moreover, to observe the impact of the number of analyzed samples on total number of identifications reported by DirectDIA we performed 12 constitutive injections, which yielded results comparable to GPF approach (Supplement 1, Figure S5 and S7A).

### Different approaches to ion-chromatogram library construction for the proteomic analysis of skeletal muscle biopsy

Subsequently, we assembled a number of ion-chromatogram libraries employing only representative fractionation and analysis type techniques or in combination with additional non-fractionated study-wide mixed sample runs (Supplement 1, Table S5), performed with DDA or DIA method. The inclusion of those non-fractionated study-wide mixed runs yielded fractionated/non-fractionated hybrid libraries, with improved retention time alignment across the fractions. Addition of those samples significantly decreased the width of extracted ion-chromatogram (XIC) windows within libraries and improved overall identification number at PSM, peptide and protein level (Supplement 1, Table S5). At this step, we selected HpH-fractionated, DDA-acquired hybrid library, supplemented with 3 non-fractionated DDA samples (HpH/DDA-H library); GPF-fractionated, STW-acquired hybrid library, supplemented with 3 non-fractionated DDA-acquired samples (GPF/STW-H library) and DirectDIA approach for subsequent analysis (Fig. 1, Supplement 1, Table S5).

**Fig. 2:**
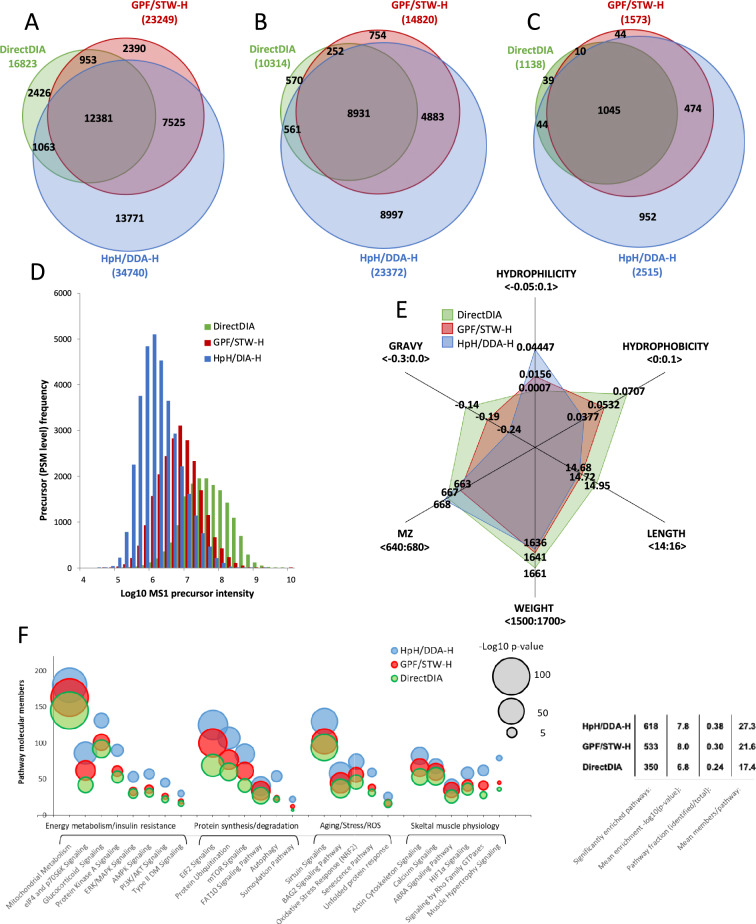
Comparison of the fractionation-based and computational-based libraries selected for the further evaluation. Venn diagrams of library overlap at precursor (Panel A), peptide (Panel B) and protein (Panel C) level; normalized histogram of precursor (peptide-spectrum match, PSM) intensity distribution (Panel D); radar plot of the peptide physicochemical properties (Panel E); pathway enrichment analysis of selected diabetes-related molecular pathways (Panel F); basic statistics of molecular pathways identified within a given ion-chromatogram library (table insert). Panel F bubble size represents significance of enrichment of a given pathway expressed as –Log10 p-value, whereas Y-axis represents the total number of identified molecular members (proteins) of a given pathway.

To further evaluate usefulness of selected approaches in the analysis of skeletal muscle proteome, we compared libraries at the level of PSMs, peptides and proteins. HpH/DDA-H hybrid library excelled in the number of unique PSMs (13,771 out of 34,740 total), which translated into highest number of unique peptides (8997 out of 23,372 total) and proteins (952 out of 2515 total) identified within this library (Fig. 2A to C, Supplement 1 Stable S5). Second-best GPF/STW-H library included 44 unique proteins (out of 1573 total), whereas DirectDIA only 39 (out of 1138 total). Both libraries were almost fully contained within HpH/DDA-H library at the level of peptides and proteins (Fig. 2A to C). HpH/DDA-H library included higher number of low-intensity PSMs (Fig. 2D), which possibly reflects peptide dilution and loss during fractionation procedure. GPF/STW-H library and DirectDIA approach yielded higher number of high-intensity PSMs, which in case of the latter could arise from prioritization of high-signal PSMs in de-novo identification pipeline and higher cut-off for low-intensity ones to decrease false-positives. At peptide physicochemical properties level, DirectDIA approach yielded slightly longer, more hydrophobic peptides, as reflected by their higher mean molecular weight, GRAVY score and hydrophobicity index as compared to both fractionation-based approaches. HpH/DDA-H library based on slightly shorter, hydrophilic peptides. This peptide characteristic could be result of chemical high-pH fractionation and retention of longer, hydrophobic peptides on ethylene bridged hybrid particles of C18 BEH column in high-pH conditions, absorption to plastic during sample transfers, and incomplete solubilization after vacuum concentration characteristic to HpH fractionation pipeline. GPF/STW-H yielded peptides with physicochemical characteristic in between those observed for DirectDIA and HpH/DDA-H regarding both the peptide length and hydrophobicity (Fig. 2E).

### Evaluation of the ion-chromatogram libraries and DirectDIA approach for the analysis of diabetes and exercise-related changes in skeletal muscle

To further evaluate usefulness of respective approaches for the analysis skeletal muscle proteome we performed pathway enrichment analysis using IPA software on proteins identified within a given library, with special emphasis on pathways connected to diabetes, muscle function and energy metabolism. IPA was able to identify 618 significantly enriched pathways in HpH/DDA-H library, covering 37% of their molecular members (pathway fraction) with mean value of 27 proteins per pathway (Fig. 2F insert). While GPF/STW-H library presented similar values to HpH/DDA-H ones, DirectDIA approach yielded considerably lower values for the number of enriched pathways, mean enrichment -log_10_(p-value), pathway fraction and mean proteins per pathway (Fig. 2F insert). Detailed enrichment analysis of insulin resistance, protein, energy metabolism, aging and muscular physiology-related pathways reflected above metrics, with HpH/DDA-H library containing highest number of proteins involved – among others—in mitochondrial metabolism, MAPK/AMPK and PI3K/AKT signaling, protein ubiquitination, autophagy, EIF2/eIF4 -controlled protein synthesis and NRF2-mediated oxidative stress response (Fig. 2F). GPF/STW-H library yielded second-best results for mitochondrial metabolism, eIF4/p70S6K signaling and protein synthesis/degradation-related pathways, whereas DirectDIA approach presented lowest number of proteins and enrichment significance in all of the analyzed pathways (Fig. 2F).

We concluded library description stage of our study with the replicate (n = 3) analysis of mixed, study-wide skeletal muscle sample to observe the impact of respective libraries on PSM, peptide and protein identification metrics. Compared to other non-hybrid library combinations, inclusion of mixed samples decreased by approx. 50% chromatographic XIC window width used for localization of MS/MS peptide fragments (from 5.1 min. to 2.9 min in case of GPF/STW-H library), which translated into higher identification scores noted for all hybrid libraries (Supplement 1, Table S6). Mean number of peptides per protein identified in mixed muscle samples ranged from 8.1 (DirectDIA) to 9 (HpH/DDA-H). Approx. 94% of proteins present in GPF/STW-H library were identified in mixed muscle samples (Library recovery, Table S6), and each mixed sample presented 92% of total number of proteins identified in triplicate (Completeness, Table S6). Peptide MS/MS spectra from GPF/STW-H library were able to explain approx. 61% of total ion chromatogram signal (Explained TIC, Table S6). For HpH/DDA-H approach, the values for library recovery, sample completeness and explained TIC equaled to 62%, 90% and 65%, respectively, whereas DirectDIA presented 100%, 100% and 61% for the above parameters. I this case, highest performance at sample completeness and library recovery can be attributed to the nature of this approach, which creates internal ion-chromatogram library from all the samples included in the experiment. This ensures the recovery of complete set of IDs from each sample, albeit with overall lower number of IDs compared to deeper, fractionation-based libraries.

**Fig. 3:**
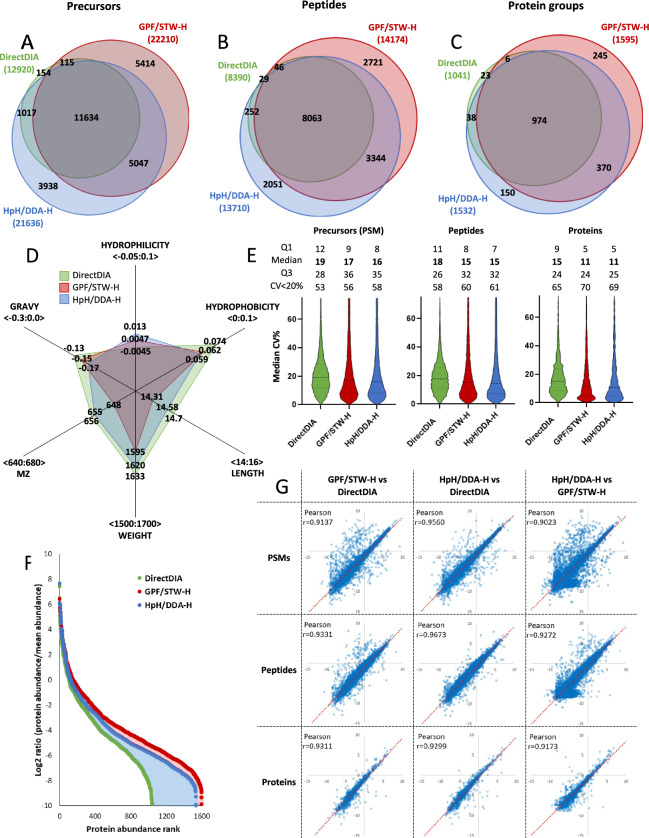
Comparison of the results of the skeletal muscle mixed sample analysis (n = 3) performed with the use of selected approaches. Venn diagrams of muscle proteins at precursor (Panel A), peptide (Panel B), and protein (Panel C) levels; radar plot of the peptide physicochemical properties (Panel D); assay precision at precursor, peptide, and protein levels (Panel E); protein rank distribution plot (Panel F); correlation plot at precursor (PSM), peptide, and protein level between the selected approaches (all correlations at *p* < 0.00001) (Panel G). Supplement 1 Figure S8 presents bubble plot of pathway enrichment analysis and basic statistics of molecular pathways identified by a given approach in the skeletal muscle mixed samples.

Surprisingly, although HpH/DDA-H outperformed other approaches as for muscular proteome coverage at library assembly stage, analysis of skeletal muscle samples with GPF/STW-H library yielded highest unique PSM, peptide and protein identifications, as compared to both the HpH/DDA-H library and DirectDIA approach (Fig. 3A to C, Table S6). Physicochemical characteristics of peptides identified within skeletal muscle samples was similar to the one observed for whole libraries, with HpH and DirectDIA displaying identification bias towards shorter, hydrophilic and longer, hydrophobic peptides, respectively (Fig. 3D). To estimate reproducibility of respective approaches, we analyzed CV% distribution at PSM, peptide and protein level. Surprisingly, most direct DirectDIA approach without fractionation and library building was characterized by highest CV% at each level of the assay, as compared to both HpH/DDA-H and GPF/STW-H (Fig. 3E). At final, protein level, median CV% equaled 15.1% for DirectDIA, 11% for GPF/STW-H, and 11% for HpH/DDA-H. Ultimately, GPF/STW-H approach yielded highest precision of the assay, with 70% of all proteins measured with CV% below 20%, compared with 69% for HpH/DDA-H and 69 for DirectDIA (Fig. 3E). Protein rank distribution analysis of respective approaches revealed that GPF/STW-H library was able to identify and quantify higher number of proteins at all abundance levels in skeletal muscle samples as compared to other approaches (Fig. 3F), with HpH/DDA-H library yielding similar, yet inferior results. Analysis performed with fractionation-based libraries gave significantly (numerically) better results regarding protein rank distribution, which was especially visible for low-abundance proteins (Fig. 3F). Quantitative measurements performed with respective approach at displayed good, significant reciprocal correlation (Pearsons r > 0.9 with *p* < 0.00001 in all cases) at both the PSM, peptide and protein level (Fig. 3G). At final, protein level best correlation was observed for GPF/STW-H vs DirectDIA data (Pearsons r = 0.9331, *p* < 0.00001), which could reflect their more direct nature of measurement, compared to chemical fractionation-based HpH/DDA-H approach of library construction (Fig. 3G). To estimate the feasibility of the particular library in the detection of the muscular proteome alterations induced by glucose intolerance, we performed pathway enrichment analysis using proteins identified by a particular approach. It revealed, that samples analyzed with GPF/STW-H library yielded highest number of significantly enriched pathways (Supplement 1, Figure S8—table insert), although pathways identified by second-best HpH/DDA-H approach displayed slightly better results regarding the mean significance of enrichment (7.8 vs 7.7 -log_10_(p-value)) and mean number of members per enriched pathway (21.4 vs 20.1). Analysis of enrichment of insulin resistance-relevant pathways revealed, that pathways from both the fractionation-based approaches had similar enrichment score (as measured by -log_10_(p-value)) and were equally populated by their molecular members (as measured by the number of identified proteins; Supplement 1, Figure S8). Pathways involved in autophagy, NRF2-mediated oxidative stress response and calcium signaling presented highest enrichment and protein count scores in HpH/DDA-H approach, whereas EIF2-mediated translation control and sirtuin signaling were most pronounced in GPF/STW-H (Supplement 1, Figure S8).

### Application of fractionation-based library approach or DirectDIA in the analysis of skeletal muscle proteomic alterations in post-training skeletal muscle from prediabetic subjects

#### Protein identification and quantitation metrics

Finally, we utilized each of the selected approaches to identify proteomic alterations invoked by glucose intolerance and insulin resistance in skeletal muscle samples from subjects which underwent structured mixed-mode exercise regimen. When applied to the whole experimental IGT/NGT sample set, both HpH/DDA-H and GPF/STW-H approaches were able to identify identical number of protein ratios (Supplement 1, Table S7, Figure S9A to C). Although DirectDIA excelled at the number of PSMs, it did not translate into higher number of peptides and proteins than those observed for both fractionation-based approaches HpH/DDA-H and GPF/STW-H (1218 vs 1456, respectively). DirectDIA displayed highest sample completeness (90% vs 64% and 84% for HpH/DDA-H and GPF/STW-H, respectively), library recovery (100% vs 86% and 99%, respectively), peptides per protein (9.8 vs 9.2 and 9.4, respectively), percentage of explained TIC (71% vs 68% and 57%, respectively) and narrowest XIC windows (3.2 min. vs 4.2 and 3.8, respectively). In most of the above quality-related determinants, GPF/STW-H presented second-best results compared to DirectDIA (Supplement 1 Table S7), simultaneously displaying similar or better numbers at PSM, peptide and protein fold change ratios than the HpH/DDA-H approach (Supplement 1 Table S7, Figure S9A to C). Comparison of identification results for proteins passing 2 unique peptides cutoff, p-value < 0.05 cutoff (equivalent to FDR-corrected Q-value of 0.05) and 50% fold change requirement (equivalent to 0.585 ≤ -log_2_(FC) ≤ -0.585), revealed that although GPF/STW-H was able to quantify greatest total number of IGT/NGT protein ratios at 2-peptide and p-value cutoff, HpH/DDA-H approach excelled in the number of unique identifications at each of the subsequent steps. Finally, HpH/DDA-H analysis yielded a total of 140 significantly affected proteins passing all of the cutoffs, compared to 131 for GPF/STW-H and 64 for DirectDIA (Supplement 1, Figure S9D to F). Protein abundance rank analysis also confirmed higher proficiency of both fractionation-based approaches in protein identification compared to DirectDIA (Supplement 1 Figure S9G), although the latter one was able to quantify 29 more IGT/NGT ratios at the lowest protein abundance compared to both the HpH/DDA-H and STW/DIA-H. Study-wide correlation analysis of protein abundance between respective approaches yielded high Pearson r values for both the IGT-only and NGT-only samples (r > 0.92, p < 0.00001 in all cases), with HpH/DDA-H and STW/DIA-H displaying highest correlation of respective protein expression (Supplement 1, Figure S10A). Correlation analysis of IGT/NGT differential protein expression (expressed as protein log2 fold change, log2FC) yielded significant (p < 0.00001) correlations between all of the approaches at the respective 2-peptide (Pearson r > 0.55, all cases) and Q-value (Pearson r > 0.7, all cases) cutoffs (Supplement 1, Figure S10B). Introduction of 50% fold-change cutoff drastically reduced the number of proteins shared between appropriate approaches, yet yielded highest Pearson r values of 0.91 and 0.96 for STW/DIA-H vs DirectDIA and HpH/DDA-H vs DirectDIA, respectively (p < 0.00001). Despite highly correlated results of protein expression between HpH/DDA-H and STW/DIA-H at the level of IGT-only and NGT-only samples, the final log2FC values for significantly affected proteins displayed modest, although significant Pearson correlation of 0.7 (Supplement 1, Figure S10B). Importantly, when compared between approaches, the shared proteins passing all 3 cutoffs and those passing 2-peptide and p-value cutoff (with minor exceptions) displayed the same direction and similar degree of differential regulation (Supplement 1, Figure S10B).

#### Molecular pathways enrichment analysis

**Fig. 4:**
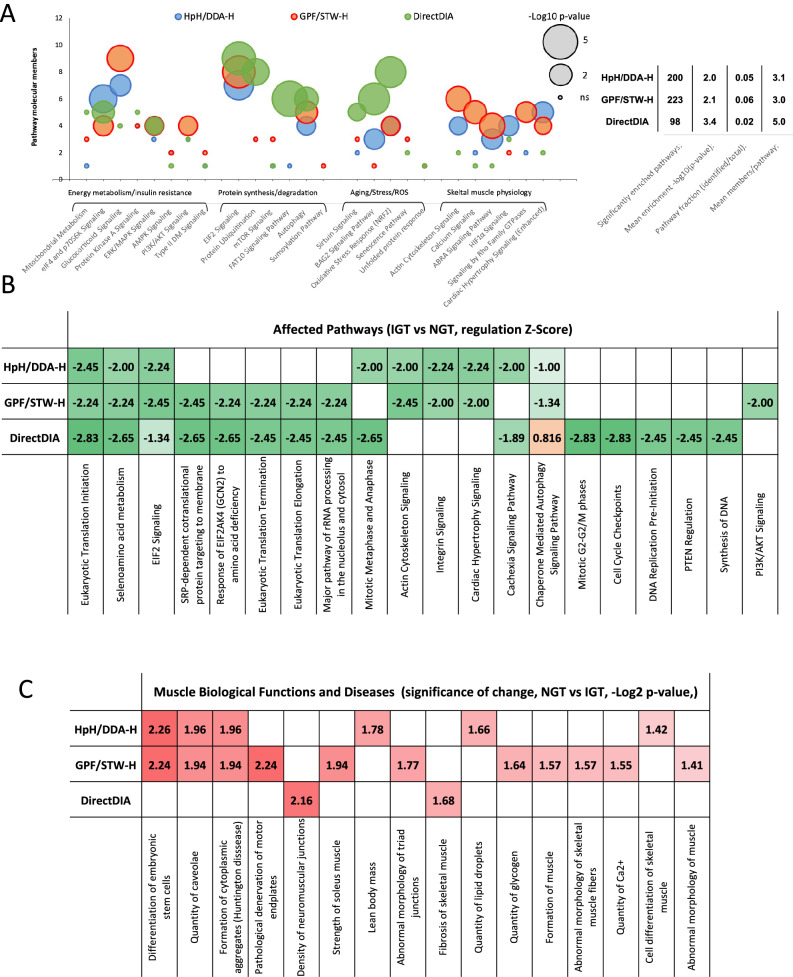
Application of the selected approaches for the analysis of post-exercise muscle proteome changes in glucose-intolerant subjects (post-exercise IGT vs NGT). Study-wide pathway enrichment analysis of selected diabetes-related molecular pathways (Panel A), with basic statistics of significantly enriched pathways (table insert); comparison of the results of pathway enrichment analysis (z-scores of regulation) showing selection of pathways relevant to preservation of healthy muscle physiology (Panel B); comparison of significantly affected skeletal muscle biological functions and potential muscular disorders (Panel C).

Subsequently, using significant-only proteins (passing all 3 cutoffs), we performed pathway enrichment analysis and protein functional clustering, to identify proteomic alterations evoked by prediabetic state in post-training muscle and to evaluate the usefulness of each approach in the identification of the above changes. Quantitatively, GPF/STW-H identified highest number of significantly affected pathways (233) compared to both HpH/DDA-H and DirectDIA (200 and 98, respectively). DirectDIA performed better than both fractionation-based approaches, regarding mean pathway enrichment and proteins/pathway metrics (Fig. 4, table insert). All of the approaches were equally effective in the detection of alterations in EIF2-mediated protein synthesis in IGT group, yet only DirectDIA was able to identify significant changes in protein ubiquitination, ubiquitin-like FAT10 signaling and detected changes in autophagy pathway with higher sensitivity than other approaches (Fig. 4A, Supplement 2 Table 6 to 8). Higher sensitivity of DirectDIA was also noted for all of the studied Aging/Stress/ROS-related pathways, such as sirtuin pathway, BAG2-mediated stress response and NRF2 oxidative stress response. Compared to both fractionation-based techniques, DirectDIA was unable to identify changes in pathways related to skeletal muscle physiology and contractile function, in which GPF/STW-H approach displayed best performance **(**Fig. 4A**)**. Interestingly, all techniques were unable to detect in IGT group alterations in molecular pathways commonly connected with insulin resistance and prediabetes, such as mitochondrial dysfunction, PKA and AMPK and Type II DM-related signaling, which suggest that above hallmarks of diabetic state were normalized in the muscle of trained prediabetics. Significant changes were observed in protein synthesis-related eIF4 and p70S6K pathways, glucocorticoid signaling, and ERK/MAPK signaling, yet only GPF/DIA-H approach was able to detect alterations in PI3K/AKT-controlled pathway.

To detect directionality of the changes, we performed z-score analysis on the significantly affected proteins, which revealed that IGT post-training muscle displayed down-regulation (z-score < 2) in a total of 7 molecular pathways responsible for control of the protein synthesis, compared to post-training NGT counterparts (selenoaminoacid metabolism, detection of amino-acid deficiency, translation initiation, elongation, termination, RNA processing and EIF2 signaling) (Fig. 4B, Supplement 2 Table 6 to 8). Both GPF/STW-H and DirectDIA were equally sensitive in the detection of the above alterations (with EIF2 pathway as an exception), whereas HPH/DDA-H approach identified only 3 out of 7 in total. Contrary to DirectDIA, both fractionation-based techniques detected significant down-regulation in the expression of proteins involved in skeletal muscle contraction (at the level of actin cytoskeleton, integrin signaling and muscle hypertrophy response, Fig. 4B), whereas only DirectDIA was able to detect detrimental alterations in pathways responsible for DNA synthesis, replication and repair and PTEN-dependent regulation of the cell cycle (Fig. 4B, Supplement 2 Table 6 to 8). Only GPF/STW-H approach was sensitive enough to detect down-regulation in the PI3K/AKT, a key insulin signaling pathway.

Regarding structural and functional alterations which distinguish post-training IGT group from their normoglycemic counterparts, both HpH/DDA-H and GPF/DIA-H methods detected greater number of changes compared to DirectDIA (Fig. 4C, Supplement 2 Table 6 to 8). Interestingly, whereas HpH/DDA-H was able to detect significant changes in lipid metabolism-related muscle functions (lean body mass retention, quantity of lipid droplets) and myofiber differentiation, GPF/DIA-H excelled in detection of all muscle contractile and morphology-related changes, such as altered Ca^2+^ storage, abnormal morphology of muscle and its fibers, strength of contraction and disrupted motor plate function (Fig. 4C, Supplement 2 Table 6 to 8). DirectDIA detected molecular alterations connected with density of neuromuscular junctions and skeletal muscle fibrosis.

#### Protein functional clustering

**Fig. 5:**
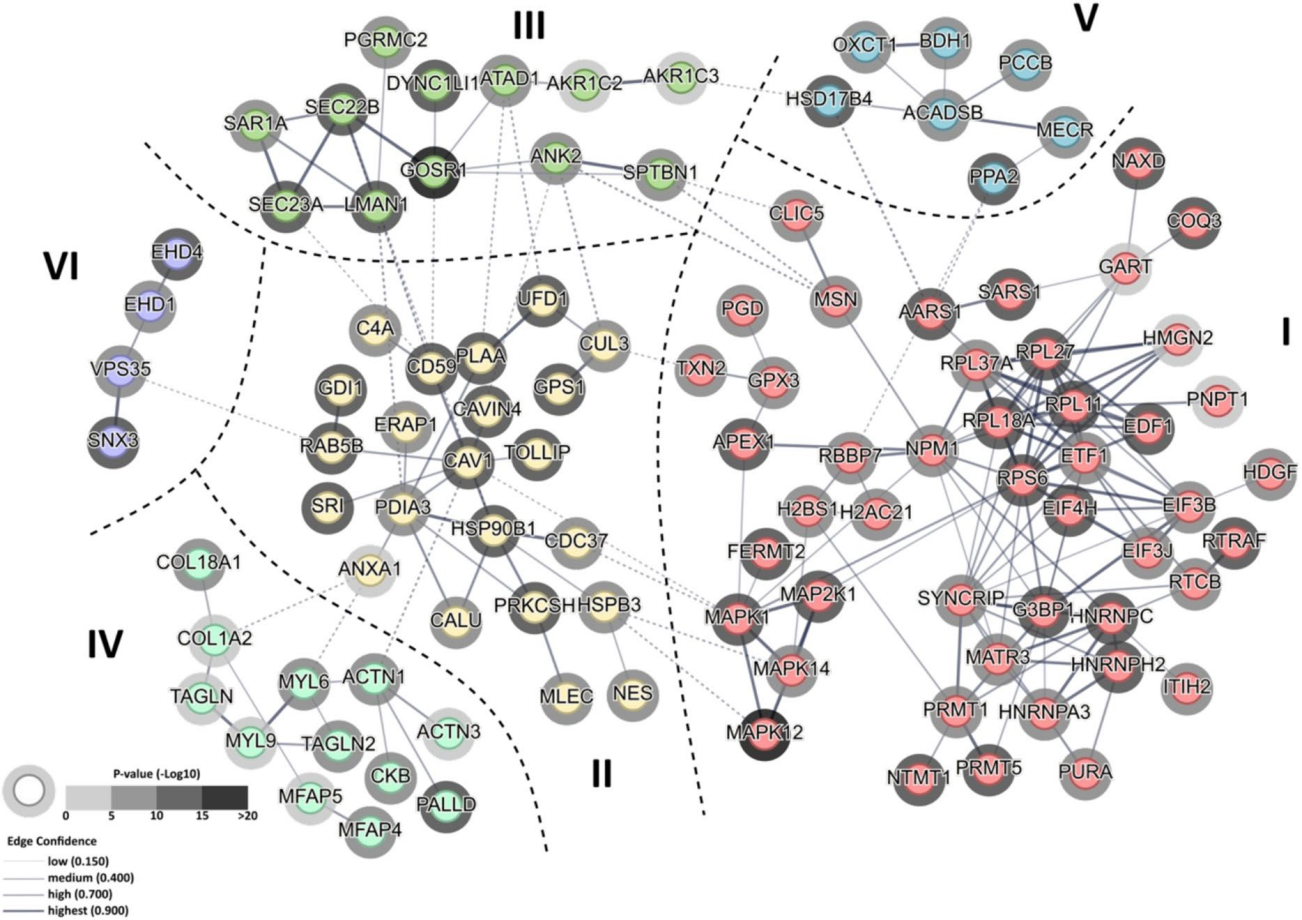
Protein functional clustering of IGT vs NGT dataset analyzed with HPH/DDA-H ion-chromatogram library. Figure shows functional clustering within 140 significantly affected proteins (orphans excluded) identified by STRING network. Analysis revealed 4 major interconnected clusters which combine proteins involved in regulation of translation and RNA processing (Cluster I), protein processing in ER (endoplasmic reticulum) (Cluster II), ER to Golgi vesicular protein transport (Cluster III) and structural constituents of skeletal muscle sarcomere (Cluster IV). Two additional clusters aggregated proteins involved in mitochondrial fatty acids metabolism (Cluster V) and endosomal protein processing (Cluster VI). Thick dashed lines denote areas occupied by clusters involved in similar biological processes. Shading of halo depicts significance of differential protein expression between NGT and IGT (expressed as –log_10_(p-value)). Line thickness indicates the strength of node interaction (edge confidence) between the members of a given cluster. Thin dashed lines represent node inter-cluster functional connections. Full results of STRING analysis are presented in Supplement 2 Table S9.

To further investigate the differences between IGT and NGT subjects observed in post-training skeletal muscle, and to identify relationships between differentially regulated proteins, we performed functional protein clustering analysis with each of the different approaches. Total of 103 out of 141 significantly affected proteins identified by HpH/DDA-H method (74% of total) generated 4 major interconnected clusters, all implicated in different aspects of protein metabolism (Fig. 5, Supplement 2, Table S9). Cluster I encompassed proteins involved in the regulation of DNA expression (MAPK kinase group), mRNA processing (HNRNP proteins), translation control (EIF factors of initiation, elongation and termination) and ribosomal subunits. Cluster II aggregated proteins involved in ER-mediated protein processing and post-translational modifications, whereas Cluster III and VI gathered proteins involved in Golgi-mediated vesicular transport and endocytic vesicular recycling. Those findings indicate that HPH/DDA-H was able to detect significant alterations in molecular control of protein synthesis, processing and trafficking in trained pre-diabetics compared to respective normoglycemic counterparts. This observation explains the presence of Cluster IV, composed of muscle contractile apparatus and extracellular matrix proteins, which suggest abnormal skeletal muscle composition in post-training IGT group compared to NGT one. Finally, HPH/DDA-H approach identified a known hallmark of prediabetes i.e. alterations in proteins connected with mitochondrial fatty acids metabolism (Cluster V).

**Fig. 6:**
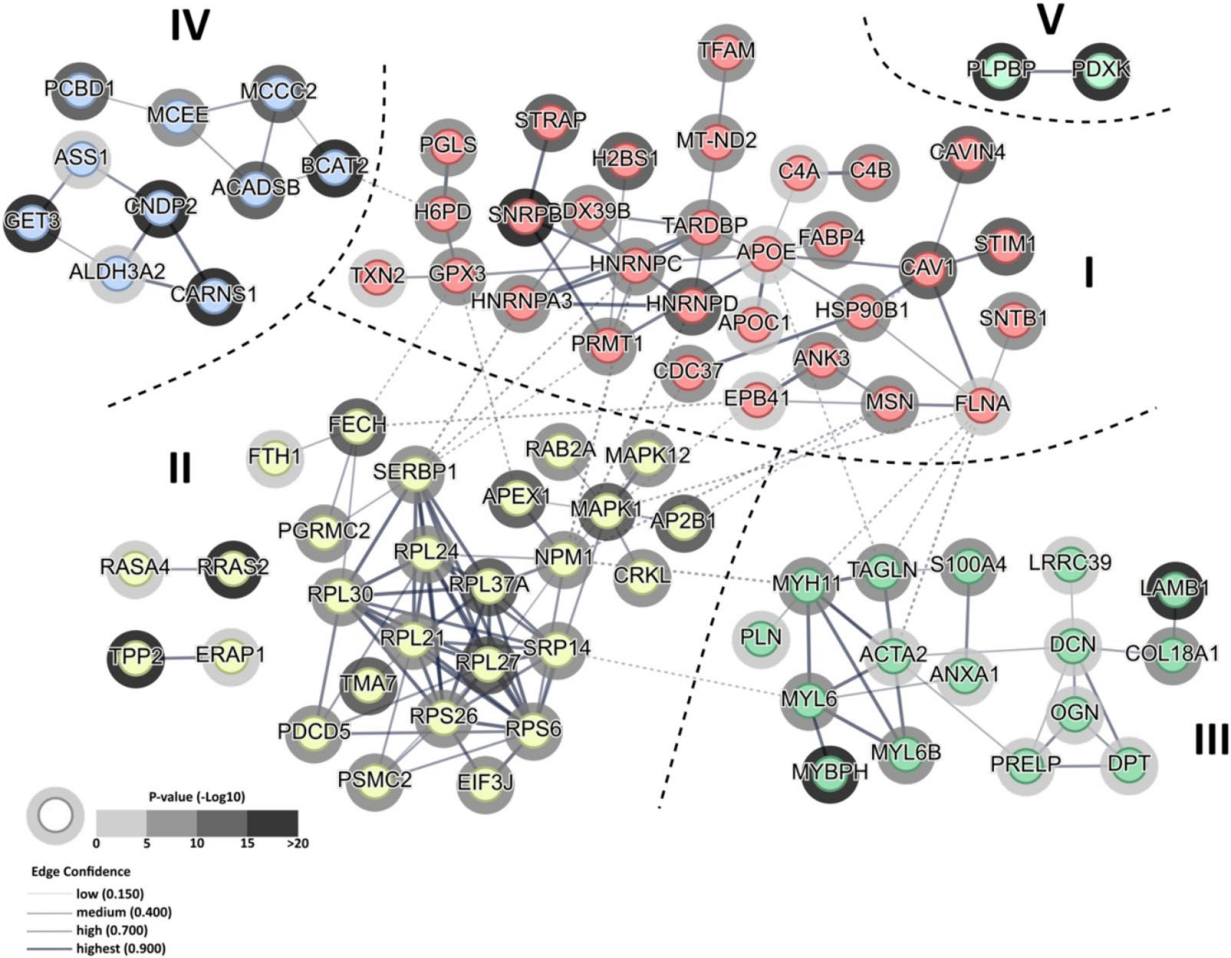
Results of protein functional clustering from IGT vs NGT dataset analyzed with GPF/STW-H ion-chromatogram library. Figure shows functional clustering within 130 significantly affected proteins (orphans excluded) identified by STRING network. Analysis revealed 3 major interconnected clusters which aggregate proteins involved in RNA splicing, lipoprotein processing and lipid uptake, and pentose phosphate pathway (Cluster I), protein synthesis, RNA binding and translation, peptide degradation and cellular proliferation (Cluster II, with 2 subclusters) and skeletal muscle contraction and myocyte-extracellular matrix interaction (Cluster III). Additional clusters combine proteins involved in branched-chain amino acid metabolism and skeletal muscle carnosine metabolism (Cluster IV, with 1 subcluster), and pyridoxal phosphate (vitamin B_6_) synthesis and transport (Cluster V). Thick dashed lines denote areas occupied by clusters involved in similar biological processes. Shading of halo depicts significance of differential protein change between NGT and IGT (expressed as -log_10_(*p*-value)). Line thickness indicates the strength of node interaction (edge confidence) between the members of a given cluster. Thin dashed lines represent node inter-cluster functional connections. Full results of STRING analysis are presented in Supplement 2 Table 10.

Similarly, clustering of 85 out of 130 (65%) of significantly affected proteins revealed by GPF/DIA-H emphasized alterations in mRNA processing, translation and protein metabolism (Cluster II and its 2 subclusters, which included MAPK kinases, EIF translation control factors, ribosomal subunits, RAS pathway members and aminopeptidases) and muscle contraction and extracellular matrix (Cluster III, which included light and heavy myosin chain isoforms, actin and EC matrix laminin B1, decorin and prolagin) (Fig. 6, Supplement 2, Table S10). Interestingly, unique finding of GFP/DIA-H approach was the identification of clusters, which molecular members are involved in branched amino-acids biosynthesis and degradation (Cluster IV e.g. mitochondrial BCAT2 branched chain aminotransferase, ACADBS short/branched chain acyl-CoA dehydrogenase, MCEE mitochondrial methylmalonyl-CoA epimerase), skeletal muscle carnosine metabolism (Cluster IV, e.g. ) and pyridoxal phosphate (vitamin B6) metabolism (Cluster V, i.e. PDXK pyridoxal kinase and PLPBP pyridoxal phosphate binding protein). Alterations in BCAA metabolism, muscular carnosine dipeptide content and vitamin B6 deficiency display strong correlation with prediabetes and subsequent progression toward Type 2 DM. Clustering of the most diverse protein set was noted for Cluster I (Fig. 6, Supplement 2, Table S10), and included proteins connected with gene expression and mRNA processing at mitochondrial and nuclear level (e.g. TFAM mitochondrial transcription factor A, proteins from HRNR heterogeneous nuclear ribonucleoprotein family, SNRBP RNA spliceosome protein and STRAP ribonucleoprotein assembly protein), cytoskeleton/plasma membrane interaction and assembly proteins (e.g. caveolae associated CAV1, CAVIN4, STIM1 proteins and cytoskeleton assembly and plasma membrane anchoring FLNA flaminin, MSN moesin, SNTB1 β-1-syntropin and ANK3 Ankyrin-3 proteins) lipid transport and metabolism-associated proteins (APOE, APOC1 lipoproteins and FABP4 fatty acids binding protein) and finally ROS metabolism and pentose phosphate pathway proteins (GPX3 glutathione peroxidase 3, TXN2 mitochondrial thioredoxin and H6PD hexose-6-phosphate dehydrogenase/glucose 1-dehydrogenase, PGLS 6-phosphogluconolactonase, respectively). APOE played the central, linking role in formation of Cluster I, possibly due to its impact on caveolae function, lipid metabolism, and mitochondrial dysfunction function (Fig. 6, Supplement 2, Table S10).

**Fig. 7:**
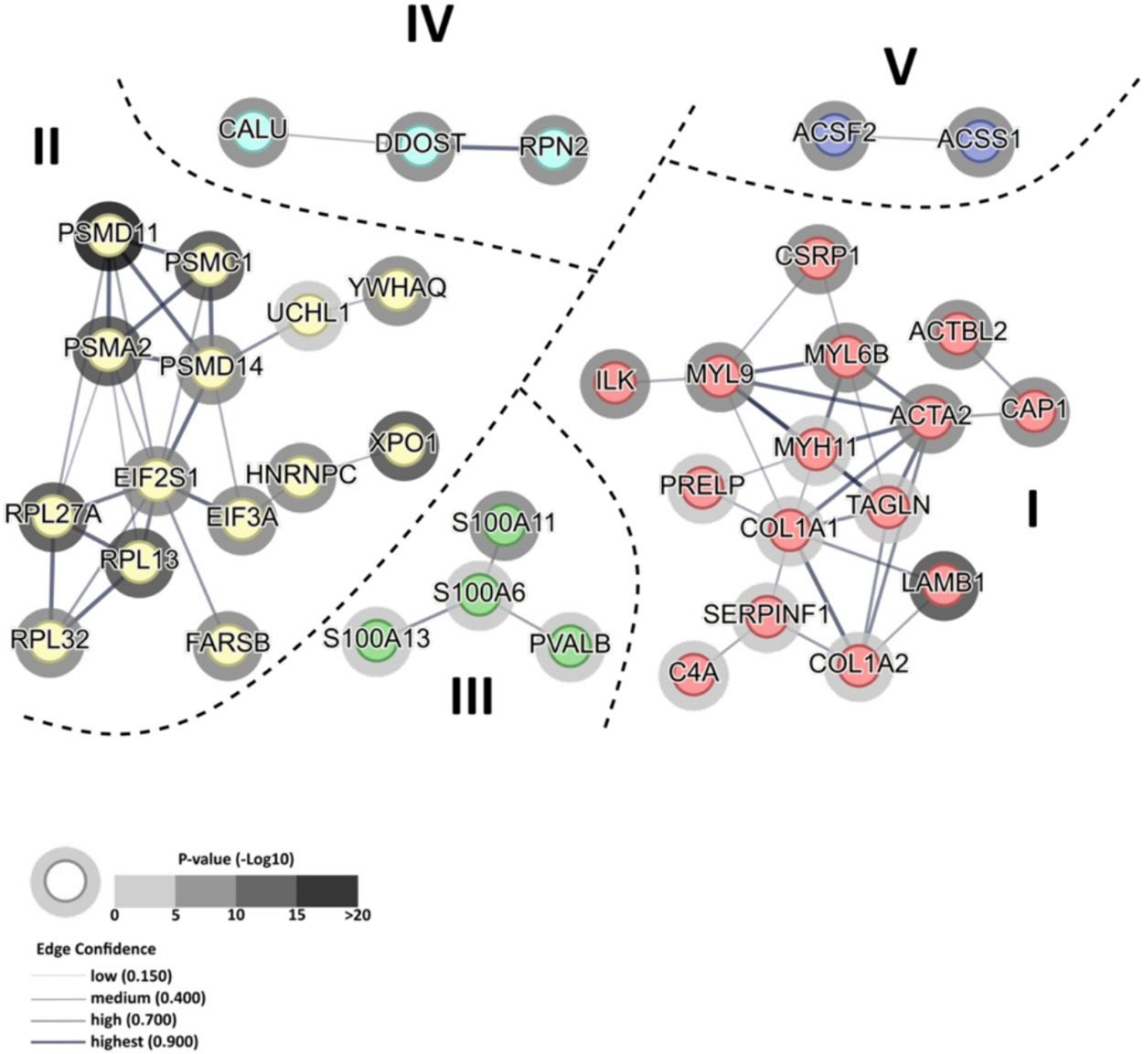
Protein functional clustering of NGT vs IGT dataset analyzed with DirectDIA approach. Figure shows functional clustering within 64 significantly affected proteins (orphans excluded) identified by STRING network. A total of 5 non-connected clusters aggregate proteins involved in muscle contraction and myocyte-intracellular matrix interaction (Cluster I), protein synthesis and proteasomal degradation (Cluster II), intra- and extracellular Ca^2+^ calcium binding (Cluster III), protein PTM (glycation) (Cluster IV), and mitochondrial medium and short-chain acyl-CoA synthesis (Cluster V). Thick dashed lines denote areas occupied by clusters involved in similar biological processes. Shading of halo depicts significance of differential protein expression between NGT and IGT (expressed as –log_10_(p-value)). Line thickness indicates the strength of node interaction (edge confidence) between the members of a given cluster. Full results of STRING analysis are presented in Supplement 2 Table 11.

Protein metabolism-related clustering of significantly affected proteins was also noted for DirectDIA analyzed IGT vs NGT dataset. A total of 38 out of 64 proteins (65%) yielded 6 independent (not connected) clusters, with Cluster I, II and IV aggregating proteins involved in muscle contraction, cytoskeleton formation and extracellular matrix (Cluster I, e.g. mysin and actin isoforms, collagens, serpin, laminin) mRNA processing, translation and protein degradation (Cluster II e.g.) and protein folding and glycosylation (Cluster V e.g. calumenin, DDOST and RPN2 glycosyltransferases). Interestingly, Cluster II included both the molecular members of protein synthesis pathways (EIF factors, ribosomal subunits) and proteasomal degradation pathway (PSM proteasomal proteins) (Fig. 7, Supplement 2, Table S11), which was unique finding of DirectDIA analysis. Cluster III connected proteins involved in muscular calcium binding and Ca^2+^ regulation of muscle contraction (S100 family proteins, parvalbumin) whereas 2 members of Cluster V shared the same molecular function as acyl-CoA – synthesizing enzymes (mitochondrial medium and short-chain Acyl-CoA ligases).

## Discussion

Introduction of high-throughput proteomic analysis in clinical applications carries a promise of possible ground-breaking discoveries at the level of both the foundational science and medical applications. Initial attempts based on 2DGE with MALDI-TOF peptide identification or FT-ICR LC/MS ultra-high resolution mass spectrometry, although encouraging from the basic science standpoint, were lacking in important qualities, such as sample throughput, reproducibility and proteome coverage. Current generation of MS-based proteomic pipelines finally resolve all the above issues. Moreover, partial application of modern pipelines on previous-generation mass spectrometers allows for the high-throughput proteomic analysis of quality and reproducibility required for clinical applications. Taking in to account all of the above, we employed modern DIA-based approach consisting of staggered-window MS/MS acquisition, customized ion-chromatogram libraries and highly reproducible semiconductor-technology based micro pillar array columns coupled with Orbitrap-HRMS for the analysis of challenging skeletal muscle samples. Fine-tuning of μPAC chromatography, DIA acquisition and library construction allowed for elucidation of skeletal muscle proteome changes in prediabetic patients, confirming presence of persistent, adverse changes in muscle proteome, despite 3 months of structured exercise.

Compared with basic science proteomics research, which can afford for multiple re-analysis attempts and longer analysis times to achieve best possible data quality, proteomics in clinical applications puts emphasis on robustness, batch-to-batch reproducibility and throughput of the complete analysis pipeline. To improve robustness and reproducibility of LC separations we employed micro pillar array columns which – compared to particle based counterparts – display decreased batch-to batch variability, significantly lower backpressures, full flow reversibility and low carryover^50^. Those features arise from semiconductor-type manufacturing process, which creates 5μm ID pillars, 18μm in height separated by 2.5μm gaps^35^. In our study, 1^st^ Gen 50cm μPAC column paired with matching 1cm μPAC trap generated 20 × lower backpressure than particle-based counterpart (25bar vs 500bar) and negligible pressure drop during trap valve operation, increasing robustness of HPLC analysis. The column displayed good retention time stability, and was resistant to clogging even in case of samples rich in fibrous biological polymers (skeletal muscle). In case of increased backpressure, μPAC column could be reverse-flushed to restore its original performance, increasing column longevity. This cannot be applied to packed, particle-based nanoflow capillary columns without the risk of stationary phase loss. Currently, pillar array columns are increasingly used in proteomics applications^36,51–54^ with 2^nd^ generation columns (2.5μm ID pillars, 25μm height 1.5μm interpillar distance) displaying improved characteristic regarding theoretical plate height and resolution^35,36^. Clinical applications can benefit from newest, rectangle pillar-based design generation (75μm x 3μm pillars), which despite its short 5.5cm length allows for higher-throughput, μLC-like analysis, with separation power comparable to its 50cm circular pillar array counterpart^51,55^.

Low column-to-column variability and retention time consistency displayed by micro pillar array column are crucial in the robust implementation of DIA analysis in both the library-based and pure bioinformatic approaches. For our study we selected staggered windows approach (STW-DIA) for both the gas-phase fractionation library generation and experimental runs, first introduced by Searle, Pino and Amodei^44–46^. Although non-overlapped window placement would yield increased MS/MS cycle times, in our view the window staggering is better suited for older-generation quadrupole-orbitrap instruments, due to the correction of non-rectangular isolation characteristic of quadrupoles and increased precision of MS/MS measurements crucial for DIA-based assays. Despite being rarely employed, staggered windows approach is gaining increasing acceptance and was used in recent studies on both previous generation and modern mass spectrometers^37,56^.

To elucidate the impact of ion-chromatogram library assemble on the results of DIA-STW based analysis of exercise-induced changes in prediabetic subjects, we selected 3 approaches with increasing deepness of the library coverage, namely High-pH fractionation (HpH), gas-phase fractionation (GPF) and pure computational approach (Spectronaut DirectDIA). Post-digestion high-pH fractionation with fraction concatenation (HpH) was shown multiple times to generate most comprehensive ion-chromatogram libraries, as compared to other protein and peptide fractionation techniques, such as PAGE, IEF focusing variants, HILIC, SCX and SAX ion exchange^57–59^. When performed at microflow ranges (50-100μl/min) with 100μg of total protein digest, we were able to perform all the necessary steps such as fraction, fraction concatenation, vacuum concentration and subsequent LC/MS/MS analysis in a single 96 well-plate format, which assures low sample loss. Nevertheless, HPLC-based physical fractionation is time-consuming, requires additional instrumentation (vacuum concentrators, dedicated HPLC with fraction collection or multimode nanoLC/microLC HPLC system etc.). In-source gas-phase fractionation offers cost and time-effective alternative to physical peptide fractionation, requiring only several additional analysis runs, albeit with lower library coverage. To further increase the deepness proteome analysis, staggered-windows gas-phase fractionation was recently combined by Penny et al. with ion-mobility technique (TIMS-diaPASEF), yielding significant improvements over its basic versions^60^. Finally, pure bioinformatic solutions to DIA analysis rely on direct identification of peptide product ions without the prior assembly of ion-chromatogram libraries. Currently, this approach to DIA analysis displays fastest growth in capabilities and multitude of available software solutions, presenting different analytical approaches (e.g. spectrum-centric, peptide-centric, in-silico fragmentation libraries etc.). Recently, pure computational DIA analysis was updated with machine learning, neural networks and AI capabilities (for excellent reviews and comparative studies see^37,61^ and^40,62^, respectively). Direct analysis of DIA-acquired MS/MS runs presents the fastest, direct approach, yet requires significant computational resources. Moreover, contrary to both the fractionation-based libraries which, ideally, should be prepared with the use of study-wide mixed sample, it can be applied continuously, in parallel to sample collection and processing before the completion of the study.

While DIA approach offers improved reproducibility and throughput, it is not without its own set of limitations. In its library-based version it typically requires the generation of high-quality spectral libraries, often built through extensive protein or peptide pre-fractionation. This is connected with additional equipment demands, increased sample quantity and processing time. Wider precursor isolation windows co-fragment multiple peptides, producing rich MS/MS spectra that require advanced deconvolution algorithms and may be subject to interference and inaccurate identification, requiring robust scoring algorithms and FDR estimation^38,63^. Moreover fragmentation efficiency and the resulting quality of product spectra depends on the correct matching of collision energy (CE) to peptide molecular size. This parameter can be fine-tuned for individual peptide precursors in DDA analysis by measuring m/z distribution of precursor isotopologue cluster ions present in survey spectra^64^. Wider Isolation windows used by DIA assays use modified CE equations and CE sweep approach which presents inferior fragmentation efficiency and produces rich MS/MS spectra decreasing reliability of identification. Finally, robust DIA approach requires high batch-to-batch retention time stability for proper library-based identification.

Taken into account all of the above, our primary objective was to identify the differences of 3 major approaches on the outcome of DIA-based analysis of muscular proteome changes evoked by exercise in prediabetic patients. We hypothesized, that assembly of spectral libraries can have direct impact on the outcome of the analysis and the final biological findings. Firstly, we selected best performing libraries among several possible combinations. Interestingly, hybrid libraries which in addition to GPF or HpH fraction runs included full mass range DDA runs from study-wide mixed samples displayed narrower XIC search windows, which translated into lower CV% of replicate analysis. Greatest improvement was noted for both of the fractionated libraries, which in our opinion is connected with the improved RT alignment of across particular library fractions, as only non-fractionated samples contained all of the proteins and peptides. Surprisingly, inclusion of non-fractionated study-wide sample had greater impact on RT alignment and XIC window width, than the presence of iRT peptides in each of the fractions, which in theory should provide RT anchor points across library runs. Although this improvement should be most visible in the case of GPF hybrid library (as iRT peptides fall in different mass fractions), it was also noted for HpH hybrid library, where iRT peptides were added to concatenated fractions prior to LC/MS runs. As expected, DirectDIA approach displayed the best RT alignment and narrowest XIC windows, yet yielded greatest CV% of replicate analysis. This outcome was somehow surprising, as protein rank distribution and precursor intensity distribution suggested, that DirectDIA displayed bias toward higher intensity signals, inherently easier to quantify. Moreover, DirectDIA analysis targeted longer, more hydrophobic peptides, compared to analysis performed with HPH/DDA-H library, which could be explained by hydrophobic peptide loss during HpH fractionation. Yet the difference was also visible between DirectDIA and GPF/STW-H analysis, which cannot be explained by mass-range gas-phase fractionation. We observed this phenomenon in both the results of the analysis of mixed-sample replicates and whole sample. Those findings suggest, that biological outcomes of the analysis performed with different approaches could differ due to targeting of peptides with different physicochemical properties, displaying slight bias toward membrane or soluble proteins.

As expected, HpH/DDA-H ion-chromatogram library contained the highest number of identified proteins and presented most populated pathways relevant to T2D and insulin resistance. Yet this advantage did not translate into higher-quality results, when employed to quantify proteins in study-wide mixed sample. The GPF/STW-H – based results were similar or better regarding quantity of proteins and their number included in T2D-relevand pathways. We hypothesize that it could be result of both the wider retention time alignment windows (Table S6, Median XIC-W: 3.7min for HpH/DDA-H vs 2.9min for GPF/STW-H vs 2.7 for DirectDIA ) and the negative effect of increased library size on overall identification rates. The HpH fractionated library displayed widest RT identification windows, which could translate into lower peptide score and identification rate. Moreover, increasing library size by extensive sample fractionation or combination of different libraries sometimes yield inferior results due to large number of low-signal peptides which cannot be identified properly when applied to experimental runs. Both factors are possibly responsible for comparable performance of HpH/DDA-H and GPF/STW-H despite HpH/DDA-H library containing significantly more members of various molecular pathways. In contrast, GPF-based libraries tended to yield more high-intensity peptides with better RT alignment which translates to more robust and uniform identification across experimental samples. This gives significant advantage for staggered-windows gas-phase fractionation approach, which requires less time and resources for implementation.

Regarding the outcome of the analysis performed on whole set of experimental samples, we noted both similarities and significant differences depending on the use of particular approach. Although we observed strong, group-wise correlations between individual proteins in IGT or NGT groups quantified with 3 different approaches (Figure S10A), the final IGT vs NGT differential expression ratios displayed weaker association (Figure S10B). Moreover, each approach turned up relatively different set of proteins, when all significance cut-offs were considered (> = 2 peptides, Q-value < 0.05, 50% FC), with only 8 common proteins (down-regulated in IGT group: TAGLN, MYBPH, KRT2, S100A13, PRELP ACADSB and up-regulated in IGT group C4A and ACTN3) common between all 3 approaches. Those discrepancies arise from differences in FDR-corrected p-values (Q-values) and calculated differential expression ratios, as those parameters substantially decreased the number of shared proteins (Figure S10B). Interestingly, both the molecular function and the direction of regulation of some of those proteins align with the metabolic and functional deficiencies observed in insulin-resistant muscle. Down-regulation of MYBPH, which is highly expressed in insulin-sensitive^65,66^, mitochondria-rich type 1 oxidative muscle fibers^67^, can reflect the decrease of fiber type in IGT group muscle, despite structured exercise regimen. Similarly, down-regulation of S100A13 calcium-binding protein, which over-expression is connected with mitochondrial biogenesis in hypoxia-trained skeletal muscle^68^, aligns with decreased oxidative capacity and mitochondrial content observed in insulin resistant muscle^69^, whereas decrease in expression of mitochondrial ACADSB branched-chain dehydrogenase can be traced to both mitochondrial deficiency^70^ and disrupted branched chain AA metabolism in prediabetic subjects^71,72^. Regarding over-expressed proteins, complement protein C4A up-regulation corresponds with increased pro-inflammatory response observed in obesity-induced insulin resistance^73,74^. Taking into account all significantly affected proteins, each approach was more sensitive towards particular changes observed in skeletal muscle proteome of IGT group. DirectDIA analysis identified pathways involved in proteasomal protein degradation, cell cycle control and DNA replication and repair, which was not observed in both fractionation-based approaches. Moreover, DirectDIA was more sensitive towards identification of changes connected with protein synthesis and degradation, oxidative stress and sirtuin signaling pathways, although all approaches signaled decreased expression of proteins involved in translation control. GPF/STW-H library based analysis was more sensitive towards detecting changes in skeletal muscle calcium signaling and morphology, whereas HpH/DDA-H in eIF4 and p70S6 kinase signaling and decreased muscle hypertrophy. Differences in biological data interpretation were also visible at the level of protein functional clustering, with DirectDIA detecting unique alternations in proteins involved in PTM glycation, Ca^2+^ binding and acyl-CoA synthesis, GPF/STW-H in proteins involved in branched amino-acid metabolism and vitamin B6 metabolism, whereas HpH/DDA-H in ER protein processing and Golgi vesicular transport. All of the approach-dependent unique protein clusters are important for metabolic function of skeletal muscle in normoglycemia, as alternations in muscular Ca^2+^ metabolism^75,76^ and protein glycation^77^, branched-chain AA^71,72^ and vitamin B6 metabolism^78,79^ and proteins-synthesis related ER stress^80,81^ are hallmarks of insulin resistance and T2DM. Nevertheless, significant alternation observed in trained IGT group as compared to respective NGT group, was the down-regulation of pathways involved in protein synthesis, despite 3 months of controlled exercise regimen. Adverse effects of insulin resistance on skeletal muscle protein synthesis could be responsible for the observed decrease in sarcomere and extracellular matrix proteins, leading to skeletal muscle contractile dysfunction^17,18^. Regarding both the qualitative and quantitative aspects of particular approach, HpH/DDA-H – based analysis identified the greatest number of significant proteins and generated unique protein interaction networks, covering important aspects of skeletal muscle metabolism such as ER stress and Golgi protein processing. On the other hand GPF/STW-H approach presented similar quantitative performance, identified significant portion of affected proteins and unique features of muscular insulin resistance (disturbance in branched AA metabolism), being less problematic to implement. Finally, libraryless DirectDIA analysis, although presented fewer significantly affected proteins, was able to robustly detect common features of skeletal muscle insulin resistance, e.g. disturbed control of protein synthesis, enhanced protein ubiquitination and proteasomal degradation and decreased DNA synthesis, replication and repair. To further explore the observation that proteasomal and DNA-metabolism proteins appeared more statistically significant in the DirectDIA dataset, we performed an in-depth comparison of the peptides underlying their identification across the three acquisition strategies. The resulting analysis (Supplement 1, Figure S11) indicates that contrary to other approaches DirectDIA preferentially quantifies slightly longer and more hydrophobic peptides, which may explain its enhanced ability to retain these proteins after statistical filtering. While significant number of these proteins are identified in all datasets (Supplement 2, Table S12), the physicochemical characteristics of the peptides may affect quantification robustness, ultimately impacting their downstream statistical significance. This phenomenon can be the result of the decreased ESI ionization efficiency^82^ and extensive CID fragmentation of hydrophobic peptides due to enlarged collisional cross-section^83^, leading to the decreased identification and quantitation scores of hydrophobic peptides.

Taking all of the above, gas-phase fractionated library, acquired in staggered windows mode and supplemented with DDA full mass range runs constitute an attractive alternative for time- and resource-consuming physical peptide fractionation. Importantly, the GPF/STW-H approach does not require extensive sample manipulation and additional offline fractionation to generate robust library, nor does it suffer from possible loss of certain peptide populations during fractionation steps. Additionally, GPF-based libraries can be easily generated multiple times during the course of longitudinal studies allowing for retrograde analysis of already surveyed samples, yielding new identifications. Finally, in our hands identification and quantitation of whole sample set using GPF/STW-H approach was computationally less demanding than pure computational approach, taking less time for the combined library building and sample analysis steps, additionally doubling the number of significant proteins. Although improvements in pure computational identification and quantitation will narrow the gap between library-based and libraryless approaches, the similar augmentation of library-based quantitation will further advance DIA-based assay. This efficiency makes GPF/STW-H approach for library generation particularly attractive for large sets, where throughput and reproducibility are critical.

## Conclusions

Among multitude of MS acquisition modes employed in proteomic assays, data-independent analysis coupled with robust chromatographic separation is best suited for large projects performed on clinical samples. Important aspect of DIA-based assay is the assembly of ion-chromatogram libraries or selection of pure computational strategy for DIA data interrogation, which hypothetically can lead distinct biological interpretations. Our goal was to employ different strategies to library construction to observe their impact on the DIA-based analysis of post-exercise differences in skeletal muscle proteome between glucose intolerant and healthy subjects. Although all tested approaches were able to detect alternations in control of protein synthesis and sarcomere protein expression, each one identified unique changes important to metabolic and contractile muscle function. In our view, staggered-windows gas-phase fractionated ion-chromatogram library presented the best balance between reproducibility, detection of significant changes, depth of analysis and ease of implementation. Combined together, the biological findings indicate, that despite extensive structured exercise regimen, insulin-resistant muscle displays disturbed molecular pathways implicated in protein synthesis, intracellular trafficking and processing, branched amino-acids and acyl-CoA mitochondrial metabolism and calcium balance, which can be the cause of muscular contractile dysfunction observed in insulin resistant subjects. Our study identified several proteins that were differentially abundant following exercise in prediabetic subjects. However, these findings were not independently validated by orthogonal methods (e.g., Western blotting, enzyme activity assays or immunofluorescence microscopy). Such validation has become a standard expectation in quantitative proteomics and is crucial for confirming changes in protein abundance and for exploring underlying molecular mechanisms. We view our proteomic analysis as an initial discovery step, and systematic follow-up validation studies are needed to confirm and functionally interpret the key findings.

## Supplementary Information

Supplementary Information 1.

Supplementary Information 2.

## Data Availability

The mass spectrometry proteomics data have been deposited to the ProteomeXchange Consortium (https://www.ebi.ac.uk/pride/archive/projects/PXD055536) via the PRIDE partner repository^84^ with the dataset identifier PXD055536.

## Abbreviations

2DPAGE: 2 Dimensional polyacrylamide gel electrophoresis
ABC: Ammonium Bicarbonate
ACG: Automatic Gain Control
ACN: Acetonitrile
AMPK: AMP-Activated Protein Kinase
AU: Arbitrary Units
AUC: Area Under the Curve
BCA: Bicinchoninic Acid
BCAA: Branched-Chain Amino Acids
BMI: Body Mass Index
Ca^2+^: Calcium Ion
Chol: Cholesterol
DDA: Data-Dependent Acquisition
DIA: Data-Independent Acquisition
DirectDIA: Direct (libraryless) Analysis of Spectra form Data-Independent Acquisition
DPP4: Dipeptidyl Peptidase 4
EIF2: Eukaryotic Initiation Factor 2
ER: Endoplasmic Reticulum
FA: Formic Acid
FDR: False Discovery Rate
FT-ICR LC/MS: Liquid chromatography/Fourier transform ion cyclotron resonance mass spectrometry
FPG: Fasting Plasma Glucose
GLP1: Glucagon-Like Peptide 1
GO: Gene Ontology
GPF: Gas-Phase Fractionation
HbA1c: Hemoglobin A1c
HDL: High-Density Lipoprotein Cholesterol
HOMA2%B: Homeostatic Model Assessment of Beta Cell Function
HOMA2%S: Homeostatic Model Assessment of Insulin Sensitivity
HOMA2-IR: Homeostatic Model Assessment for Insulin Resistance
HOMAD: Homeostatic Model Assessment for Diabetes
HpH: Reverse-phase HPLC Peptide Fractionation in high-pH conditions
HRMS: High-Resolution Mass Spectrometry
IAA: Iodoacetamide
IFG: Impaired Fasting Glucose
IGT: Impaired Glucose Tolerance
INS: Insulin
iRT: Stable Isotope-labelled peptides for Peptide Indexed Retention Time calculation
iTRAQ: Isobaric Tag for Relative and Absolute Quantitation
LC/MS/MS: Liquid Chromatography/Tandem Mass Spectrometry
LDL: Low-Density Lipoprotein Cholesterol
LN_2_: Liquid Nitrogen
MALDI-TOF: Matrix-assisted laser desorption/ionization – time of flight mass spectrometry
MSX: Multiplexed MS/MS
NCE: Normalized Collision Energy
NGT: Normal Glucose Tolerance
OGTT: Oral Glucose Tolerance Test
PSM: Peptide-Spectrum Match
PTM/PTMs: Post-Translational Modification/Post-Translational Modifications
SDC: Sodium Deoxycholate
SGLT2: Sodium-Glucose Co-Transporter 2
SMM: Skeletal Muscle Mass
STRING: Search Tool for the Retrieval of Interacting Genes/Proteins
STW: Staggered Windows
T2DM: Type 2 Diabetes Mellitus
TCEP: Tris(2-carboxyethyl)phosphine
TFA: Trifluoroacetic Acid
TG: Triglycerides
TIC: Total Ion Chromatogram
TMT: Tandem mass tags
VAT_DXA: Visceral Adipose Tissue measured by Dual-Energy X-ray Absorptiometry
VO_2_max: Maximal Oxygen Uptake
μPAC: Micro Pillar Array Column

## References

[1] Voeltz, D. et al. Projecting the economic burden of type 1 and type 2 diabetes mellitus in Germany from 2010 until 2040. *Popul. Health Metr.* **22**, 17 (2024). 39026351 10.1186/s12963-024-00337-x PMC11264726

[2] Parker, E. D. et al. Economic Costs of Diabetes in the U.S. in 2022. *Diabetes Care* **47**, 26–43 (2024). 37909353 10.2337/dci23-0085

[3] ElSayed, N. A. et al. Diagnosis and Classification of Diabetes: Standards of Care in Diabetes-2024. *Diabetes Care* **47**, S20–S42 (2024). 38078589 10.2337/dc24-S002 PMC10725812

[4] Dludla, P. V. et al. Pancreatic β-cell dysfunction in type 2 diabetes: Implications of inflammation and oxidative stress. *World J. Diabetes* **14**, 130–146 (2023). 37035220 10.4239/wjd.v14.i3.130 PMC10075035

[5] Mezza, T. et al. β-Cell Fate in Human Insulin Resistance and Type 2 Diabetes: A Perspective on Islet Plasticity. *Diabetes* **68**, 1121–1129 (2019). 31109941 10.2337/db18-0856 PMC6905483

[6] DeMarsilis, A. et al. Pharmacotherapy of type 2 diabetes: An update and future directions. *Metabolism* **137**, 155332 (2022). 36240884 10.1016/j.metabol.2022.155332

[7] Pantalone, K. M. et al. Clinical characteristics, complications, comorbidities and treatment patterns among patients with type 2 diabetes mellitus in a large integrated health system. *BMJ Open Diabetes Res. Care* **3**, e000093 (2015). 26217493 10.1136/bmjdrc-2015-000093 PMC4513350

[8] Galaviz, K. I. et al. Interventions for Reversing Prediabetes: A Systematic Review and Meta-Analysis. *Am. J. Prev. Med.* **62**, 614–625 (2022). 35151523 10.1016/j.amepre.2021.10.020 PMC10420389

[9] Borges-Canha, M. et al. Prediabetes remission after bariatric surgery: a 4-years follow-up study. *BMC Endocr. Disord.* **24**, 7 (2024). 38200480 10.1186/s12902-024-01537-0 PMC10782579

[10] Zhang, H. et al. Exercise training modalities in prediabetes: a systematic review and network meta-analysis. *Front. Endocrinol. (Lausanne).* **15**, 1308959 (2024). 38440785 10.3389/fendo.2024.1308959 PMC10911289

[11] Mirmiran, P., Hosseini, S., Bahadoran, Z. & Azizi, F. Dietary pattern scores in relation to pre-diabetes regression to normal glycemia or progression to type 2 diabetes: a 9-year follow-up. *BMC Endocr. Disord.* **23**, 20 (2023). 36670395 10.1186/s12902-023-01275-9 PMC9854100

[12] Strain, T. et al. Quantifying the Relationship Between Physical Activity Energy Expenditure and Incident Type 2 Diabetes: A Prospective Cohort Study of Device-Measured Activity in 90,096 Adults. *Diabetes Care* **46**, 1145–1155 (2023). 36693275 10.2337/dc22-1467 PMC10234747

[13] Yang, W. et al. Different levels of physical activity and risk of developing type 2 diabetes among adults with prediabetes: a population-based cohort study. *Nutr. J.* **23**, 107 (2024). 39289701 10.1186/s12937-024-01013-4 PMC11406853

[14] Furlano, J. A., Horst, B. R., Petrella, R. J., Shoemaker, J. K. & Nagamatsu, L. S. Changes in cognition and brain function after 26 weeks of progressive resistance training in older adults at risk for diabetes: A pilot randomized controlled trial. *Can. J. Diabetes* **47**, 250–256 (2023). 36858923 10.1016/j.jcjd.2023.01.004

[15] Luvuno, M., Khathi, A. & Mabandla, M. V. The effects of exercise treatment on learning and memory ability, and cognitive performance in diet-induced prediabetes animals. *Sci. Rep.* **10**, 15048 (2020). 32929110 10.1038/s41598-020-72098-0 PMC7490284

[16] Lu, H.-H., Zhou, Y., Chen, C. & Gu, Z.-J. Meta-analysis of the effect of exercise intervention on cognitive function in elderly patients with type 2 diabetes mellitus. *BMC Geriatr.* **24**, 770 (2024). 39300333 10.1186/s12877-024-05352-z PMC11411744

[17] Senefeld, J. W., Harmer, A. R. & Hunter, S. K. Greater lower limb fatigability in people with prediabetes than controls. *Med. Sci. Sports Exerc.* **52**, 1176–1186 (2020). 31815831 10.1249/MSS.0000000000002238

[18] Dlamini, M. & Khathi, A. Investigating the effects of diet-induced prediabetes on skeletal muscle strength in male sprague dawley rats. *Int. J. Mol. Sci.* **25**, 4076 (2024). 38612885 10.3390/ijms25074076 PMC11012655

[19] Ripley, E. M. et al. Reduced skeletal muscle phosphocreatine concentration in type 2 diabetic patients: a quantitative image-based phosphorus-31 MR spectroscopy study. *Am. J. Physiol. Endocrinol. Metab.* **315**, E229–E239 (2018). 29509433 10.1152/ajpendo.00426.2017 PMC6139498

[20] Fabbri, E. et al. Insulin resistance is associated with reduced mitochondrial oxidative capacity measured by 31P-magnetic resonance spectroscopy in participants without diabetes from the baltimore longitudinal study of aging. *Diabetes* **66**, 170–176 (2017). 27737951 10.2337/db16-0754 PMC5204309

[21] Houzelle, A. et al. Human skeletal muscle mitochondrial dynamics in relation to oxidative capacity and insulin sensitivity. *Diabetologia* **64**, 424–436 (2021). 33258025 10.1007/s00125-020-05335-w PMC7801361

[22] Szczerbinski, L. et al. The response of mitochondrial respiration and quantity in skeletal muscle and adipose tissue to exercise in humans with prediabetes. *Cells* **10**, 3013 (2021). 34831236 10.3390/cells10113013 PMC8616473

[23] Deshmukh, A. S. et al. Deep muscle-proteomic analysis of freeze-dried human muscle biopsies reveals fiber type-specific adaptations to exercise training. *Nat. Commun.* **12**, 304 (2021). 33436631 10.1038/s41467-020-20556-8 PMC7803955

[24] Kjærgaard, J. et al. Insulin- and exercise-induced phosphoproteomics of human skeletal muscle identify REPS1 as a regulator of muscle glucose uptake. *Cell Reports. Med.* **6**, 102163 (2025). 10.1016/j.xcrm.2025.102163 PMC12208332 40482643

[25] Larsen, J. K. et al. Illumination of the Endogenous Insulin-Regulated TBC1D4 Interactome in Human Skeletal Muscle. *Diabetes* **71**, 906–920 (2022). 35192682 10.2337/db21-0855 PMC9074744

[26] Ohlendieck, K. Skeletal muscle proteomics: current approaches, technical challenges and emerging techniques. *Skelet. Muscle* **1**, 6 (2011). 21798084 10.1186/2044-5040-1-6 PMC3143904

[27] Cervone, D. T., Moreno-Justicia, R., Quesada, J. P. & Deshmukh, A. S. Mass spectrometry-based proteomics approaches to interrogate skeletal muscle adaptations to exercise. *Scand. J. Med. Sci. Sports* **34**, e14334 (2024). 36973869 10.1111/sms.14334

[28] Gonzalez-Freire, M. et al. The human skeletal muscle proteome project: A reappraisal of the current literature. *J. Cachexia Sarcopenia Muscle* **8**, 5–18 (2017). 27897395 10.1002/jcsm.12121 PMC5326819

[29] Hesketh, S. J., Stansfield, B. N., Stead, C. A. & Burniston, J. G. The application of proteomics in muscle exercise physiology. *Expert Rev. Proteomics* **17**, 813–825 (2020). 33470862 10.1080/14789450.2020.1879647

[30] Fernández-Costa, C. et al. Impact of the Identification Strategy on the Reproducibility of the DDA and DIA Results. *J. Proteome Res.* **19**, 3153–3161 (2020). 32510229 10.1021/acs.jproteome.0c00153 PMC7898222

[31] Bekker-Jensen, D. B. et al. Rapid and site-specific deep phosphoproteome profiling by data-independent acquisition without the need for spectral libraries. *Nat. Commun.* **11**, 787 (2020). 32034161 10.1038/s41467-020-14609-1 PMC7005859

[32] Shen, B., Pade, L. R. & Nemes, P. Data-independent acquisition shortens the analytical window of single-cell proteomics to fifteen minutes in capillary electrophoresis mass spectrometry. *J. Proteome Res.* **24**, 1549–1559 (2025). 39325989 10.1021/acs.jproteome.4c00491 PMC11936843

[33] Barkovits, K. et al. Reproducibility, specificity and accuracy of relative quantification using spectral library-based data-independent acquisition. *Mol. Cell. Proteomics* **19**, 181–197 (2020). 31699904 10.1074/mcp.RA119.001714 PMC6944235

[34] Kjærgaard, J. et al. Personalized molecular signatures of insulin resistance and type 2 diabetes. *Cell*10.1016/j.cell.2025.05.005 (2025). 40436015 10.1016/j.cell.2025.05.005

[35] Vankeerberghen, B., de Beeck, J. O. & Desmet, G. On-chip comparison of the performance of first- and second-generation micropillar array columns. *Anal. Chem.* **95**, 13822–13828 (2023). 37677150 10.1021/acs.analchem.3c01829

[36] Stejskal, K. et al. Deep proteome profiling with reduced carryover using superficially porous microfabricated nanolc columns. *Anal. Chem.* **94**, 15930–15938 (2022). 36356180 10.1021/acs.analchem.2c01196 PMC9685595

[37] Fröhlich, K. et al. Data-independent acquisition: A milestone and prospect in clinical mass spectrometry-based proteomics. *Mol. Cell. Proteomics* **23**, 100800 (2024). 38880244 10.1016/j.mcpro.2024.100800 PMC11380018

[38] Searle, B. C. et al. Generating high quality libraries for DIA MS with empirically corrected peptide predictions. *Nat. Commun.* **11**, 1548 (2020). 32214105 10.1038/s41467-020-15346-1 PMC7096433

[39] Govaert, E. et al. Comparison of fractionation proteomics for local SWATH library building. *Proteomics* **17**, 1700052 (2017). 28664598 10.1002/pmic.201700052 PMC5601298

[40] Lou, R. et al. Benchmarking commonly used software suites and analysis workflows for DIA proteomics and phosphoproteomics. *Nat. Commun.* **14**, 94 (2023). 36609502 10.1038/s41467-022-35740-1 PMC9822986

[41] Szczerbinski, L. et al. Metabolomic profile of skeletal muscle and its change under a mixed-mode exercise intervention in progressively dysglycemic subjects. *Front. Endocrinol. (Lausanne).* **12**, 778442 (2021). 34938272 10.3389/fendo.2021.778442 PMC8685540

[42] Shanely, R. A. et al. Human skeletal muscle biopsy procedures using the modified Bergström technique. *J. Vis. Exp.*10.3791/51812 (2014). 25285722 10.3791/51812 PMC4828068

[43] León, I. R., Schwämmle, V., Jensen, O. N. & Sprenger, R. R. Quantitative assessment of in-solution digestion efficiency identifies optimal protocols for unbiased protein analysis. *Mol. Cell. Proteomics* **12**, 2992–3005 (2013). 23792921 10.1074/mcp.M112.025585 PMC3790306

[44] Searle, B. C. et al. Chromatogram libraries improve peptide detection and quantification by data independent acquisition mass spectrometry. *Nat. Commun.* **9**, 5128 (2018). 30510204 10.1038/s41467-018-07454-w PMC6277451

[45] Pino, L. K., Just, S. C., MacCoss, M. J. & Searle, B. C. Acquiring and analyzing data independent acquisition proteomics experiments without spectrum libraries. *Mol. Cell. Proteomics* **19**, 1088–1103 (2020). 32312845 10.1074/mcp.P119.001913 PMC7338082

[46] Amodei, D. et al. Improving precursor selectivity in data-independent acquisition using overlapping windows. *J. Am. Soc. Mass Spectrom.* **30**, 669–684 (2019). 30671891 10.1007/s13361-018-2122-8 PMC6445824

[47] Bruderer, R. et al. Optimization of experimental parameters in data-independent mass spectrometry significantly increases depth and reproducibility of results. *Mol. Cell. Proteomics* **16**, 2296–2309 (2017). 29070702 10.1074/mcp.RA117.000314 PMC5724188

[48] Krämer, A., Green, J., Pollard, J. J. & Tugendreich, S. Causal analysis approaches in ingenuity pathway analysis. *Bioinformatics* **30**, 523–530 (2014). 24336805 10.1093/bioinformatics/btt703 PMC3928520

[49] Szklarczyk, D. et al. The STRING database in 2023: protein-protein association networks and functional enrichment analyses for any sequenced genome of interest. *Nucleic Acids Res.* **51**, D638–D646 (2023). 36370105 10.1093/nar/gkac1000 PMC9825434

[50] Rozing, G. Micropillar array columns for advancing nanoflow HPLC. *Microchem. J.* **170**, 106629 (2021).

[51] Matzinger, M. et al. Micropillar arrays, wide window acquisition and AI-based data analysis improve comprehensiveness in multiple proteomic applications. *Nat. Commun.* **15**, 1019 (2024). 38310095 10.1038/s41467-024-45391-z PMC10838342

[52] Stejskal, K., de Beeck, J. O., Dürnberger, G., Jacobs, P. & Mechtler, K. Ultrasensitive NanoLC-MS of Subnanogram Protein Samples Using Second Generation Micropillar Array LC Technology with Orbitrap Exploris 480 and FAIMS PRO. *Anal. Chem.* **93**, 8704–8710 (2001). 10.1021/acs.analchem.1c00990 PMC8253486 34137250

[53] Cho, B. G., Jiang, P., Goli, M., Gautam, S. & Mechref, Y. Using micro pillar array columns (μPAC) for the analysis of permethylated glycans. *Analyst* **146**, 4374–4383 (2021). 34132263 10.1039/d1an00643f

[54] Berg, H. E. et al. Micro-pillar array columns (µPAC): An efficient tool for comparing tissue and cultured cells of glioblastoma. *J. Chromatogr. Open* **2**, 100047 (2022).

[55] Vankeerberghen, B., de Beeck, J. O. & Desmet, G. Column-Only Band Broadening in a Porous Shell Radially Elongated Pillar Array Column. *Anal. Chem.* **96**, 3618–3626 (2024). 38350649 10.1021/acs.analchem.3c05756

[56] Doellinger, J., Blumenscheit, C., Schneider, A. & Lasch, P. Isolation Window Optimization of Data-Independent Acquisition Using Predicted Libraries for Deep and Accurate Proteome Profiling. *Anal. Chem.* **92**, 12185–12192 (2020). 32840101 10.1021/acs.analchem.0c00994

[57] Batth, T. S. & Olsen, J. V. Offline High pH Reversed-Phase Peptide Fractionation for Deep Phosphoproteome Coverage. *Methods Mol. Biol.* **1355**, 179–192 (2016). 26584926 10.1007/978-1-4939-3049-4_12

[58] Yang, F., Shen, Y., Camp, D. G. 2nd. & Smith, R. D. High-pH reversed-phase chromatography with fraction concatenation for 2D proteomic analysis. *Expert Rev. Proteomics* **9**, 129–134 (2012). 22462785 10.1586/epr.12.15 PMC3337716

[59] Yeung, D. et al. Separation Orthogonality in Liquid Chromatography-Mass Spectrometry for Proteomic Applications: Comparison of 16 Different Two-Dimensional Combinations. *Anal. Chem.* **92**, 3904–3912 (2020). 32030975 10.1021/acs.analchem.9b05407

[60] Penny, J., Arefian, M., Schroeder, G. N., Bengoechea, J. A. & Collins, B. C. A gas phase fractionation acquisition scheme integrating ion mobility for rapid diaPASEF library generation. *Proteomics* **23**, e2200038 (2023). 36876969 10.1002/pmic.202200038

[61] Kitata, R. B., Yang, J.-C. & Chen, Y.-J. Advances in data-independent acquisition mass spectrometry towards comprehensive digital proteome landscape. *Mass Spectrom. Rev.* **42**, 2324–2348 (2023). 35645145 10.1002/mas.21781

[62] Gotti, C. et al. Extensive and Accurate Benchmarking of DIA Acquisition Methods and Software Tools Using a Complex Proteomic Standard. *J. Proteome Res.* **20**, 4801–4814 (2021). 34472865 10.1021/acs.jproteome.1c00490

[63] Lou, R. & Shui, W. Acquisition and Analysis of DIA-Based Proteomic Data: A Comprehensive Survey in 2023. *Mol. Cell. Proteomics* **23**, 100712 (2024). 38182042 10.1016/j.mcpro.2024.100712 PMC10847697

[64] Révész, Á. et al. Tailoring to search engines: Bottom-up proteomics with collision energies optimized for identification confidence. *J. Proteome Res.* **20**, 474–484 (2021). 33284634 10.1021/acs.jproteome.0c00518 PMC7786379

[65] Stuart, C. A. et al. Slow-twitch fiber proportion in skeletal muscle correlates with insulin responsiveness. *J. Clin. Endocrinol. Metab.* **98**, 2027–2036 (2013). 23515448 10.1210/jc.2012-3876 PMC3644602

[66] Albers, P. H. et al. Human muscle fiber type-specific insulin signaling: Impact of obesity and type 2 diabetes. *Diabetes* **64**, 485–497 (2015). 25187364 10.2337/db14-0590

[67] Murgia, M. et al. Protein profile of fiber types in human skeletal muscle: A single-fiber proteomics study. *Skelet. Muscle* **11**, 24 (2021). 34727990 10.1186/s13395-021-00279-0 PMC8561870

[68] Lanfranchi, C. et al. Repeated sprint training in hypoxia induces specific skeletal muscle adaptations through S100A protein signaling. *FASEB J.* **38**, e23615 (2024). 38651657 10.1096/fj.202302084RR

[69] Sangwung, P., Petersen, K. F., Shulman, G. I. & Knowles, J. W. Mitochondrial Dysfunction, Insulin Resistance, and Potential Genetic Implications. *Endocrinology* **161**, (2020). 10.1210/endocr/bqaa017 PMC7341556 32060542

[70] Ruegsegger, G. N., Creo, A. L., Cortes, T. M., Dasari, S. & Nair, K. S. Altered mitochondrial function in insulin-deficient and insulin-resistant states. *J. Clin. Invest.* **128**, 3671–3681 (2018). 30168804 10.1172/JCI120843 PMC6118582

[71] Newgard, C. B. Interplay between lipids and branched-chain amino acids in development of insulin resistance. *Cell Metab.* **15**, 606–614 (2012). 22560213 10.1016/j.cmet.2012.01.024 PMC3695706

[72] Zhang, H. et al. Branched-chain amino acid supplementation impairs insulin sensitivity and promotes lipogenesis during exercise in diet-induced obese mice. *Obesity (Silver Spring)* **30**, 1205–1218 (2022). 35357085 10.1002/oby.23394

[73] Wu, C., Xu, G., Tsai, S.-Y.A., Freed, W. J. & Lee, C.-T. Transcriptional profiles of type 2 diabetes in human skeletal muscle reveal insulin resistance, metabolic defects, apoptosis, and molecular signatures of immune activation in response to infections. *Biochem. Biophys. Res. Commun.* **482**, 282–288 (2017). 27847319 10.1016/j.bbrc.2016.11.055

[74] Ajjan, R. A. & Schroeder, V. Role of complement in diabetes. *Mol. Immunol.* **114**, 270–277 (2019). 31400630 10.1016/j.molimm.2019.07.031

[75] Eshima, H. Influence of Obesity and Type 2 Diabetes on Calcium Handling by Skeletal Muscle: Spotlight on the Sarcoplasmic Reticulum and Mitochondria. *Front. Physiol.* **12**, 758316 (2021). 34795598 10.3389/fphys.2021.758316 PMC8592904

[76] Sun, G., Vasdev, S., Martin, G. R., Gadag, V. & Zhang, H. Altered calcium homeostasis is correlated with abnormalities of fasting serum glucose, insulin resistance, and beta-cell function in the Newfoundland population. *Diabetes* **54**, 3336–3339 (2005). 16249463 10.2337/diabetes.54.11.3336

[77] Du, H. et al. Advanced glycation end products induce skeletal muscle atrophy and insulin resistance via activating ROS-mediated ER stress PERK/FOXO1 signaling. *Am. J. Physiol. Endocrinol. Metab.* **324**, E279–E287 (2023). 36724125 10.1152/ajpendo.00218.2022

[78] Liu, Z. et al. Vitamin B6 Prevents Endothelial Dysfunction, Insulin Resistance, and Hepatic Lipid Accumulation in Apoe (-/-) Mice Fed with High-Fat Diet. *J. Diabetes Res.* **2016**, 1748065 (2016). 26881239 10.1155/2016/1748065 PMC4735993

[79] Mascolo, E. & Vernì, F. Vitamin B6 and Diabetes: Relationship and Molecular Mechanisms. *Int. J. Mol. Sci.* **21**, (2020). 10.3390/ijms21103669 PMC7279184 32456137

[80] Cheng, F. et al. Exercise activates autophagy and regulates endoplasmic reticulum stress in muscle of high-fat diet mice to alleviate insulin resistance. *Biochem. Biophys. Res. Commun.* **601**, 45–51 (2022). 35228120 10.1016/j.bbrc.2022.02.058

[81] Flamment, M., Hajduch, E., Ferré, P. & Foufelle, F. New insights into ER stress-induced insulin resistance. *Trends Endocrinol. Metab.* **23**, 381–390 (2012). 22770719 10.1016/j.tem.2012.06.003

[82] Bagag, A. et al. Characterization of hydrophobic peptides in the presence of detergent by photoionization mass spectrometry. *PLoS ONE* **8**, e79033 (2013). 24236085 10.1371/journal.pone.0079033 PMC3827311

[83] Meier, F. et al. Deep learning the collisional cross sections of the peptide universe from a million experimental values. *Nat. Commun.* **12**, 1185 (2021). 33608539 10.1038/s41467-021-21352-8 PMC7896072

[84] Perez-Riverol, Y. et al. The PRIDE database resources in 2022: a hub for mass spectrometry-based proteomics evidences. *Nucleic Acids Res.* **50**, D543–D552 (2022). 34723319 10.1093/nar/gkab1038 PMC8728295

