# Supplementary material for: The impact of exercise on skeletal muscle proteome of prediabetic subjects analyzed with data independent mass spectrometry
Source: Sci Rep. 2025 Aug 7;15:28988. doi: 10.1038/s41598-025-13942-z (PMC12331922; doi:10.1038/s41598-025-13942-z)
Supplement: Supplementary file 1 — Supplementary Information 1. [file 41598_2025_13942_MOESM1_ESM.pdf]

Title: The impact of exercise on skeletal muscle proteome of prediabetic subjects analyzed with data independent mass spectrometry

This article contains the following supplemental data:

Supplement_1.pdf

Table S1. Post-exercise characteristics of studied groups.

Table S2. Optimization of µPAC column chromatography gradient.

Table S3. Gradient time and flow optimization of µPAC column.

Table S4. Sample load optimization of µPAC column.

Table S5. Basic statistics of ion-chromatogram libraries.

Table S6. Analysis of mixed human muscle biopsy samples (n=3) performed with the use of different ion-chromatogram library combinations and DirectDIA.

Table S7. Analysis of study-wide muscle biopsy samples (n=48) performed with the use of respective ion-chromatogram libraries and DirectDIA approach.

Figure S1. Retention time stability and FWHM of sample-spiked iRT peptides and total ion chromatograms (TIC) of experimental samples.

Figure S2. Comparison of HPLC column pressure for particle-based and pillar array-based chromatography columns.

Figure S3. Stability of MS and MS/MS mass measurement of sample-spiked iRT peptides and peptides from experimental samples.

Figure S4. MS/MS level signal normalization of samples analyzed with selected ion-chromatogram libraries and DirectDIA approach.

Figure S5. Results of gas-phase fractionation of ions (GPF) for library generation.

Figure S6. Results of off-line high-pH fractionation (HpH) of peptides for library generation.

Figure S7. Peptide/protein identifications using different approaches for the generation of ion-chromatogram library.

Figure S8. Pathway enrichment analysis of selected diabetes-related molecular pathways and basic statistics of molecular pathways identified with a given ion-chromatogram library and DirectDIA approach (table insert).

Figure S9. Comparison of the results of IGT vs NGT differential expression analysis at various stages of protein identification and quantitation.

Figure S10. Correlation analysis of protein expression from IGT vs NGT comparison.

Figure S11. Radar plot of the peptide physicochemical properties from significantly affected proteasomal degradation-related proteins.

Supplement_2.xlsx

Table_S1_24mz STW (Sample) – Staggered windows placement for sample acquisition method.

Table_S2_4mz STW (GPF) – Staggered windows placement for gas-phase fractionation method.

Table_S3_IPA_Pathways – Comparison of results - affected pathways (IPA analysis).

Table_S4_IPA_Dissease_Bio_Funct – Comparison of results – diseases and bio-functions (IPA analysis).

Table_S5_IPA_Molecules – Comparison of results – identified proteins and their IGT/NGT differential expression ratios (IPA analysis).

Table_S6_HpH_IPA_Report – Full IPA report of dataset analyzed with HpH ion-chromatogram library.

Table_S7_GPF_IPA_Report – Full IPA report of dataset analyzed with GPF ion-chromatogram library.

Table_S8_DirectDIA_IPA_Report – Full IPA report of dataset analyzed with DirectDIA approach.

Table_S9_HpH_STRING – Results of protein functional clustering performed on dataset analyzed with HpH ion-chromatogram library.

Table_S10_GPF_STRING – Results of protein functional clustering performed on dataset analyzed with GPF ion-chromatogram library.

Table_S11_DirectDIA_STRING – Results of protein functional clustering performed on dataset analyzed with DirectDIA approach.

Table_S12 - Proteasomal degradation-related proteins identified as significantly affected by the appropriate approach in IGT/NGT comparison.

Table S1. Post-exercise characteristics of the studied groups. NGT - normal fasting glucose and normal glucose tolerance; IGT – impaired fasting glucose and impaired glucose tolerance; HbA1c – Haemoglobin A1c; OGTT AUC – oral glucose tolerance test area under plasma glucose concentration curve; INS AUC – oral glucose tolerance test area under plasma insulin concentration curve; HOMA2 (%B) – homeostatic model assessment of beta cell function; HOMA2 (%S) – homeostatic model assessment of insulin sensitivity; HOMA2-IR - homeostatic model assessment for insulin resistance; BMI – body mass index; SMM – skeletal muscle mass; Total_Bone_Mass – bone mass; Total_Fat_Mass – body fat mass (both the surface level and internal fat); Total_Lean_Mass – fat-free mass; VAT_DXA – dual-energy X-ray absorptiometry (DXA)-derived visceral adipose tissue (VAT); Chol – cholesterol; TG – triglycerides; HDL – high-density lipoprotein cholesterol; LDL – low-density lipoprotein cholesterol; VO_2max – maximal oxygen consumption.

	NGT	IGT
Age (y)	48.00±7.11	48.64±8.45
Weight (kg)	88.00±9.32	98.21±13.20
HbA1c (%)	5.07±0.39	5.46±0.23*
Fasting glucose (mg/dl)	99.38±5.28	111.82±6.05*
Fasting insulin (mIU/L)	9.60±4.23	22.09±11.03*
OGTT AUC (AU)	24670±4712	34175±4533*
INS AUC (AU)	13433±6098	29982±11839*
HOMA2 %B	89.56±28.09	127.52±51.42*
HOMA2 %S	94.15±42.84	41.75±18.83*
HOMA2 IR	1.27±0.56	2.92±1.37*
BMI	27.62±3.35	32.40±4.73*
SMM (kg)	36.72±2.25	37.02±4.37
Body fat (kg)	22.91±7.43	32.75±10.02*
Total_Bone_Mass (kg)	3.11±0.48	3.09±0.38
Total_Fat_Mass (kg)	25.29±6.97	33.15±7.81*
Total_Lean_Mass (kg)	59.74±4.49	61.85±6.47
VAT_DXA(kg)	1.57±0.65	2.64±1.05*
Chol	195.6±38.2	212.3±31.2
TG	91.8±36.4	161.0±66.2*
HDL	53.08±13.63	53.55±15.65
LDL	132.7±38.6	142.1±22.3
VO_2 max (ml/min/kg)	34.52±5.17	29.77±3.01*
VO_2 max (ml/min)	3022.5±434.1	2908.9±373.0

Values are mean ± SD (NGT n = 13; IGT n=11); *-p < 0.05 vs. NGT; significance by Mann Whitney *U* test.

Table S2. Optimization of µPAC column chromatography gradient. All runs were performed in triplicate on 200ng of HeLa protein digest standard in Top 20 DDA mode as described in Materials and Methods. Column flow rate 300nl/min.

	**µPAC column gradient**		
	**G1 (55min standard)**	**G2 (85min linear)**	**G3 (85min non-linear)**
#MS	12604	17645	16299
#MS/MS	41837	72496	77983
#PSMs	26224	41611	44399
#Peptides	10587	16114	17174
#Sequences	10189	14961	16023
#Protein Groups	1849	2461	2534
#Proteins	2240	2827	2917

Gradient G1 (55min standard) – 4%B-4min, 25%B-35min, 40%B-45min, 90%B (1ul/min)-46min, 90%B (1ul/min)-49min, 4%B-50min, 4%B-55min.
Gradient G2 (85min linear) – 4%B-4min, 45%B-64min, 90%B-65min, 90%B-75min, 1.1%B-76min, 5%B-85min.
Gradient G3 (85min non-linear) – 4%B-4min, 30%B-49min, 45%B-64min, 90%B-65min, 90%B-75min, 5%B-76min, 4%B-85min.
A-0.2% FA; B-90%ACN 0.2%FA.
Values are cumulative totals from triplicate runs (n=3).

Table S3. Gradient time and flow optimization of µPAC column. Gradient steps across different run times were normalized proportionately to the total length of the gradient, except initial trap loading time. Non-linear optimized G3 gradient was used as the starting point. Runs were performed in triplicate on 200ng of HeLa protein digest standard in Top 20 DDA mode as described in Materials and Methods.

	**µPAC column gradient**			
	**G3 (45min) 500nl/min**	**G3 (55min) 400nl/min**	**G3 (85min) 300nl/min**	**G3 (115min)* 300nl/min**
#MS	10565	13686	16299	18511
#MS/MS	51828	64017	77983	81800
#PSMs	35238	41922	44399	50518
#Peptides	13490	15625	17174	18660
#Sequences	12822	14736	16023	17217
#Protein Groups	2283	2513	2534	2762
#Proteins	2649	2917	2917	3203

G3-Gradient: 5%B-4min, 30%B-71min, 45%B-94min, 90%B-95min, 90%B-103min, 5%B-104min, 5%B-115min.
A-0.2% FA; B-90%ACN 0.2%FA.
* - selected gradient length/flow settings.
Values are cumulative totals from triplicate runs (n=3).

Table S4. Sample load optimization of µPAC column. Runs were performed in triplicate on HeLa protein digest standard. Mass spectrometer operated in Top 20 DDA mode as described in Materials and Methods. Column flow rate 300nl/min.

	**µPAC column gradient**			
	**G3 (115min) 250ng**	**G3 (115min) 500ng**	**G3 (115min) * 750ng**	**G3 (115min) 1000ng**
#MS	21207	20146	19832	19380
#MS/MS	120337	125167	126635	128413
#PSMs	66171	69769	71090	71066
#Peptides	23016	23780	24199	24505
#Sequences	20944	21485	21682	21839
#Protein Groups	3423	3482	3609	3616
#Proteins	3887	3912	4058	4060

G3-Gradient: 5%B-4min, 30%B-71min, 45%B-94min, 90%B-95min, 90%B-103min, 5%B-104min, 5%B-115min
A-0.2% FA; B-90%ACN 0.2%FA.
* - selected column load setting.
Values are cumulative totals from triplicate runs (n=3).

Table S5. Basic statistics of ion-chromatogram libraries. Individual ion-chromatogram libraries were created from mixed human muscle biopsy samples analyzed with gas-phase fractionation (GPF, 4m/z STW), full mass-range staggered-windows method (24m/z STW) and high-pH fractionation with fraction concatenation using full mass-range DDA (Top 20) or STW (24m/z STW) method (HpH/DDA and HpH/STW, respectively), as described in Materials and Methods. Hybrid libraries were supplemented with additional 3 independent runs performed with full mass-range Top 20 DDA or 24m/z STW method.

	**Ion-chromatogram libraries – library statistics**							
	**GPF/STW**	**GPF/STW (+3xSTW) HYBRID**	**GPF/STW * (+3xDDA) HYBRID**	**HpH/DDA**	**HpH/STW**	**HpH/STW (+3xSTW) HYBRID**	**HpH/DDA * (+3xDDA) HYBRID**	**DirectDIA * (6xSTW)**
#Fragments	108950	118819	133902	193493	127966	140008	202576	96564
#PSMs	19406	21109	23249	33370	22978	24953	34740	16823
#Peptides	15343	16540	14820	26880	18037	19529	23372	10314
#Sequences	11722	11974	13609	21354	14018	14533	21664	9466
#Protein Groups	1620	1605	1573	2600	1763	1807	2515	1138
#Proteins	1717	1690	1666	2691	1860	1913	2607	1213
#Single Hits	375	336	259	499	372	386	454	224

GPF/STW – 6 gas-phase fractions (4m/z GPF-STW method).
GPF/STW (+3xSTW) HYBRID – 6 gas-phase fractions (4m/z STW) supplemented with 3 full-mass range STW runs (24m/z STW).
GPF/STW (+3xDDA) HYBRID – 6 gas-phase fractions (4m/z STW) supplemented with 3 full-mass range DDA runs (Top 20 DDA).
HpH/DDA – 6 high-pH concatenated fractions (Top 20 x DDA method).
HpH/STW – 6 high-pH concatenated fractions (24m/z STW).
HpH/STW (+3xSTW) HYBRID - 6 high-pH concatenated fractions (Top 20 x DDA) supplemented with 3 full-mass range STW runs (24m/z STW).
HpH/DDA (+3xDDA) HYBRID - 6 high-pH concatenated fractions (Top 20 x DDA) supplemented with 3 full-mass range DDA runs (Top 20 x DDA).
DirectDIA (6xSTW) – library-less DirectDIA assay from 6 independent STW runs (24m/z STW).
* - selected for further evaluation.
Values as given by Spectronaut (Library perspective)

Table S6. Analysis of mixed human muscle biopsy samples (n=3) performed with the use of different ion-chromatogram library combinations and DirectDIA approach. Mixed muscle biopsy samples were analyzed with full-mass range 24m/z STW as described in Materials and Methods. Subsequently, different ion-chromatogram libraries were used for protein identification.

	Ion-chromatogram libraries – sample identification							
	**GPF/STW**	**GPF/STW (+3xSTW) HYBRID**	**GPF/STW * (+3xDDA) HYBRID**	**HpH/DDA**	**HpH/STW**	**HpH/STW (+3xSTW) HYBRID**	**HPH/DDA * (+3xDDA) HYBRID**	**DirectDIA * (3xSTW)**
#PSMs	18408	20332	22210	20194	18837	22736	21636	12920
#Peptides	14608	15958	17521	16499	14958	18540	17084	10364
#Sequences	12113	12565	14174	14007	12683	15378	13710	8390
#Protein Groups	1453	1488	1595	1358	1324	1469	1532	1041
#Proteins	1539	1567	1665	1409	1412	1528	1601	1115
#Peptides/Protein	7.9	8.1	8.6	8.8	8.6	8.8	9	8.1
Explained TIC	50%	52%	61%	62%	64%	63%	65%	61%
Completeness	93%	94%	92%	86%	90%	86%	90%	100.0%
Lib. Recovery	95%	96%	94%	61%	82%	64%	62%	100.0%
Median CVs (pep)	21%	21%	15%	13%	13%	14%	15%	18%
Median CVs (prot)	17%	17%	11%	10%	11%	10%	11%	15%
Median XIC-W (min.)	5.1	3	2.9	5.2	5.6	3.9	3.9	2.7
Median DPPP	8.3	8.3	8.3	8.3	8.3	8.3	8.3	8.3

***** - final selection, referred in manuscript as GPF hybrid approach, HpH hybrid approach and DirectDIA approach, respectively.
Values are sparse profiles from triplicate runs (n=3) as given by Spectronaut (Post-analysis perspective).
Median XIC-W – median window width used for the localization of the peptide extracted ion chromatograms (XIC) - does not correspond to FWHM.
DPPP - data points per peak (full width), as measured at FWHM*1.7.

Table S7. Analysis of study-wide muscle biopsy samples (n=48) performed with the use of respective ion-chromatogram libraries and DirectDIA approach. Study samples were analyzed with full-mass range 24m/z STW as described in Materials and Methods. Subsequently, best-performing approaches were used for protein identification.

	Ion-chromatogram libraries (whole sample set)		
	HPH/DDA-H	GPF/STW-H	DirectDIA
#PSMs	19071	20210	22272
#Peptides	15635	16087	17777
#Sequences	13177	13164	11562
#Protein Groups	1465	1465	1218
#Proteins	1526	1554	1282
#Peptides/Protein	9.2	9.4	9.8
Explained TIC	68%	57%	71%
Completeness	64%	84%	90%
Lib. Recovery	86%	99%	100%
Median CVs (pep)	38%	36%	32%
Median CVs (prot)	33%	33%	31%
Median XIC-W (min.)	4.2	3.8	3.2
Median DPPP	9.2	9.2	9.8

Values are 0.5 percentile sparse profiles (proteins present in at least 50% of the samples) from whole sample set, as given by Spectronaut (Post-analysis perspective).
Median XIC-W – median window width used for the localization of the peptide extracted ion chromatograms (XIC) - does not correspond to FWHM.
DPPP - data points per peak (full width), as measured at FWHM*1.7.

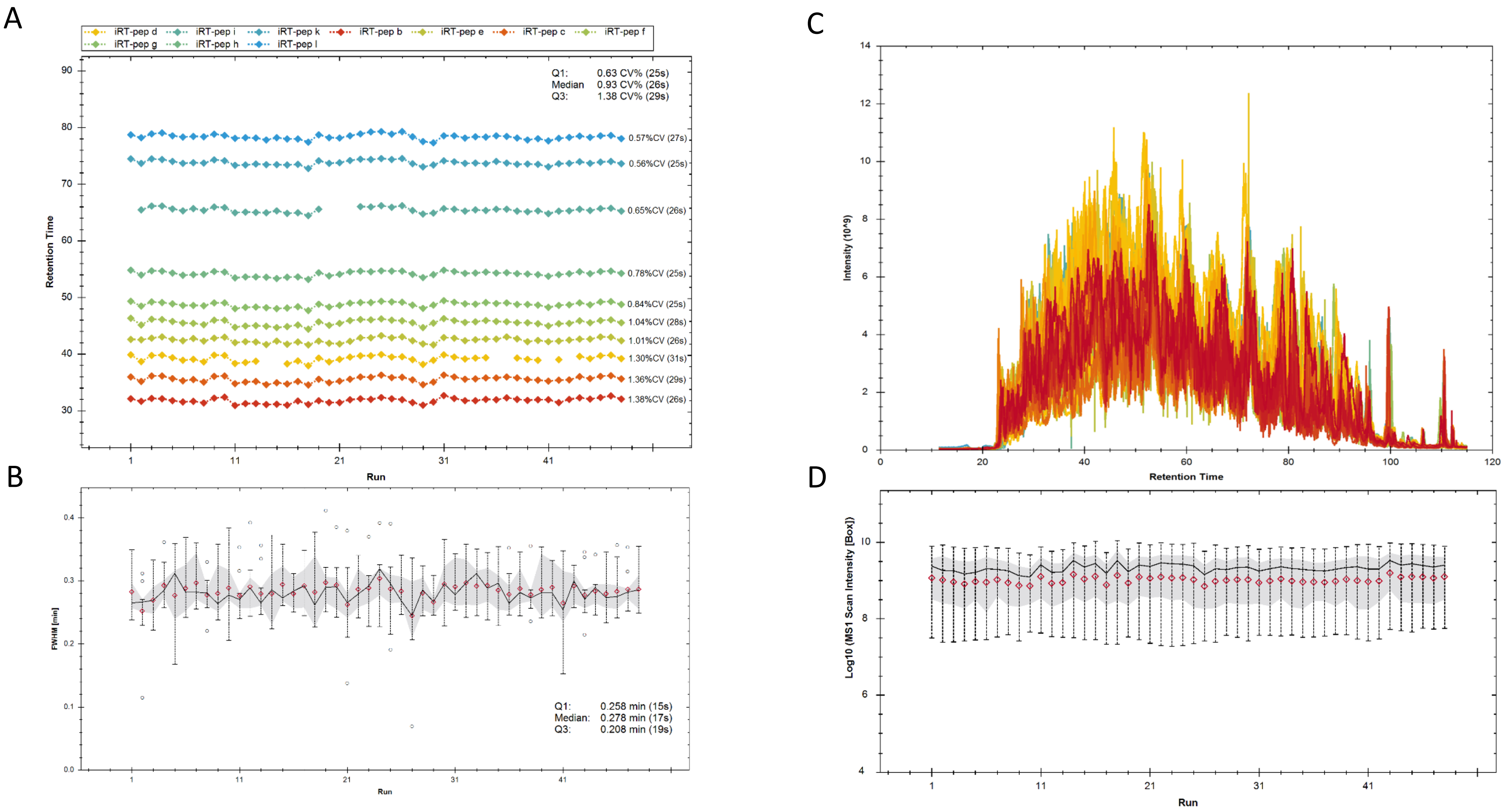

Figure S1. Retention time stability of sample-spiked iRT peptides and total ion chromatograms (TIC) of experimental samples. Panel A – Retention time variation of individual iRT peptides. **Panel B** – Full width at half medium (FWHM) values of sample-spiked iRT peptides. **Panel C –** Overlay of total ion chromatograms (TIC) of 48 experimental samples. **Panel D** – MS1 level detector response of experimental samples.

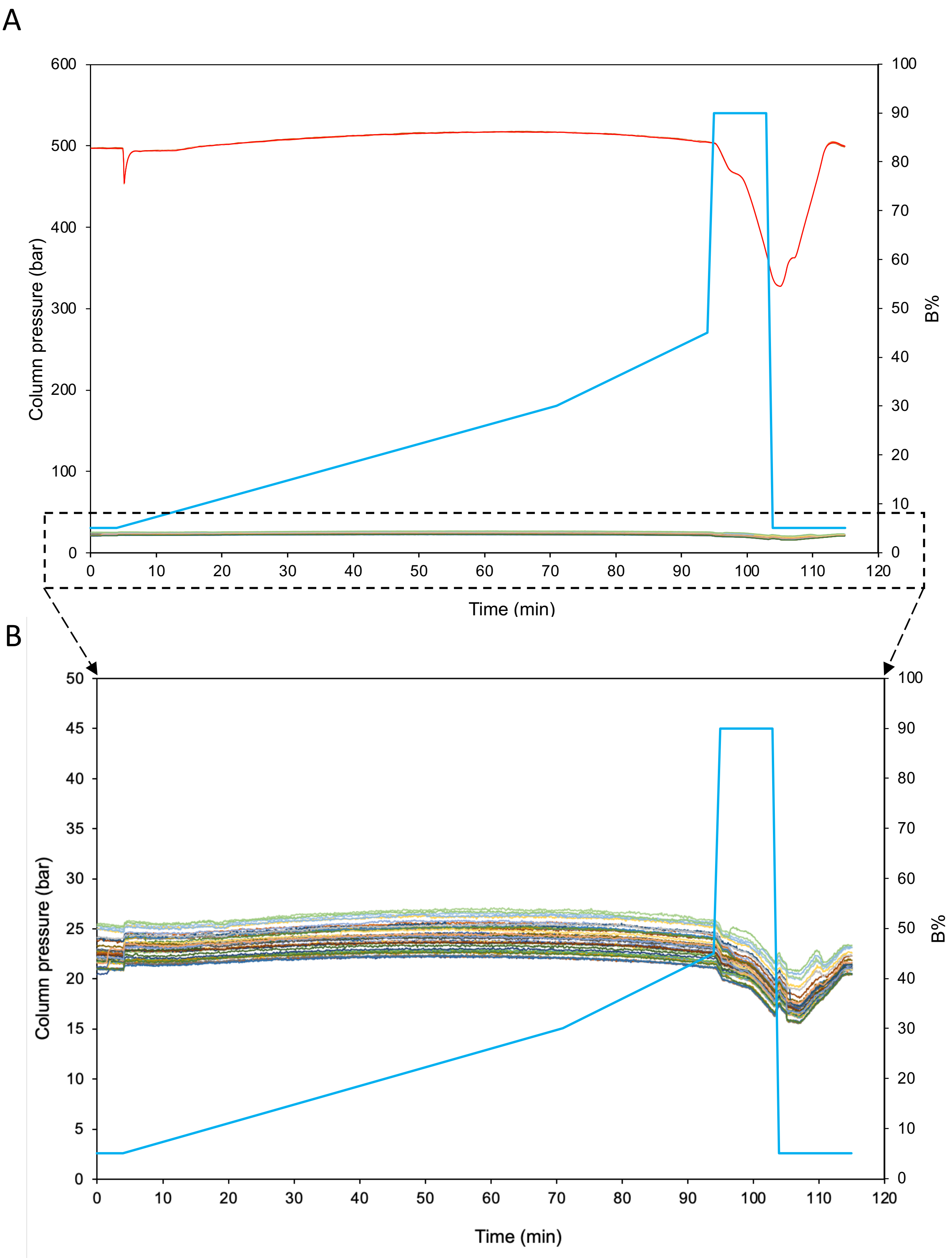

Figure S2. Comparison of HPLC column pressure for particle-based and pillar array-based chromatography columns. Panel A – Pressure plot of 50cm capillary nanoflow column (50cm dl, 75µm ID, 2µm grain, C18). **Panel B** – Pressure plots of 48 consecutive skeletal muscle sample runs performed on 50cm µPAC column (channel length 500mm, channel width 315µm, pillar height 18µm, pillar diameter 5µm, interpillar distance 2.5µm). Both columns operated under identical 115min gradient time and shape (blue plot, right axis), flow rates (300nl/min) and mobile phases (A-0.2% FA in H_2O; B-90%ACN, 0.2%FA in H_2O).

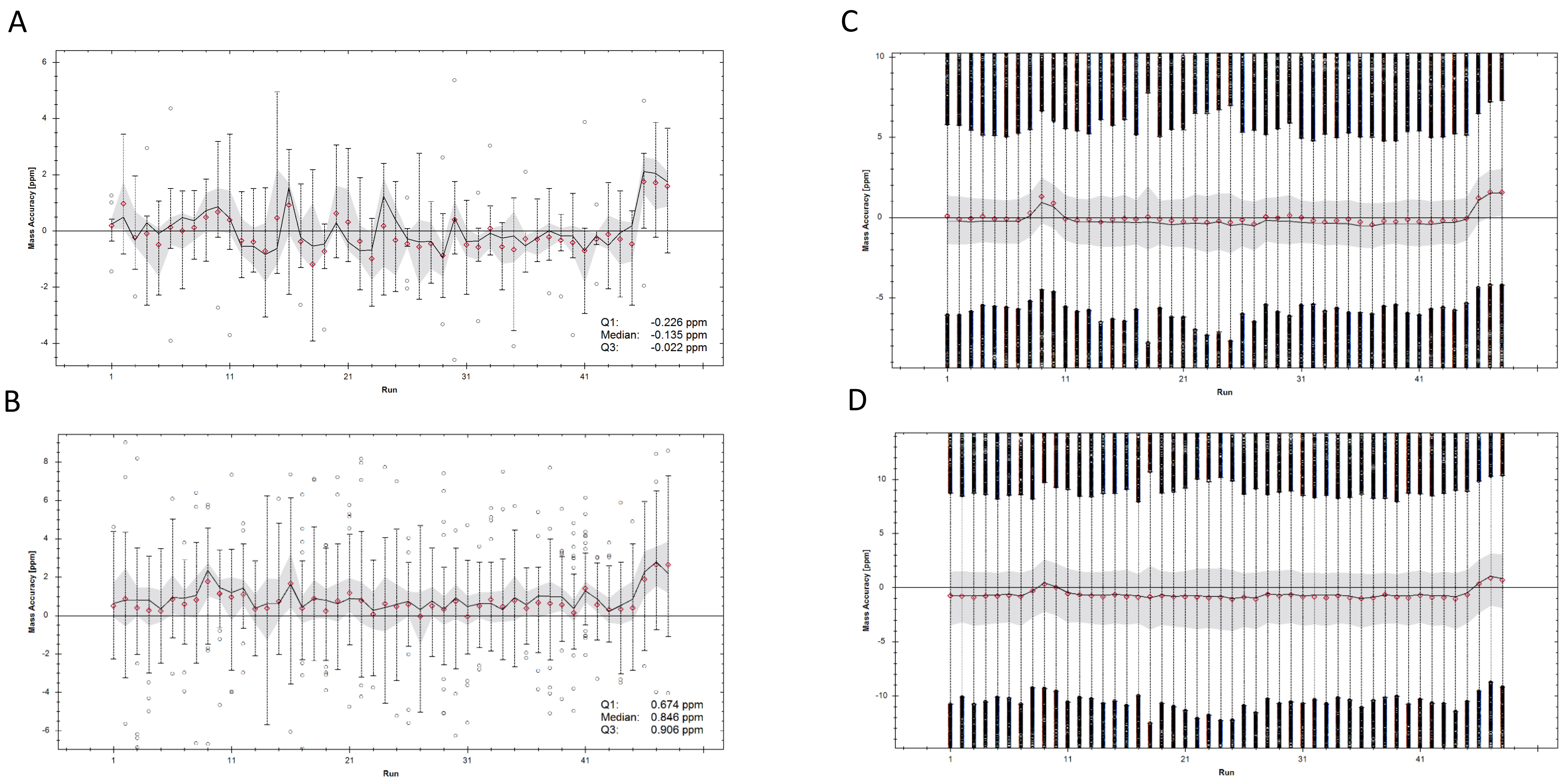

Figure S3. Stability of MS and MS/MS mass measurement of sample-spiked iRT peptides and peptides from experimental samples. Panel A - Accuracy of full MS mass measurement of sample-spiked iRT peptides. **Panel B** – Accuracy of MS/MS mass measurement of product ions of sample-spiked iRT peptides. **Panel C** – Accuracy of full MS mass measurement of peptides from all experimental samples. **Panel D** – Accuracy of MS/MS mass measurement of product ions of peptides from all experimental samples. Analysis was performed using staggered windows 24m/z STW method as described in Materials and Methods section.

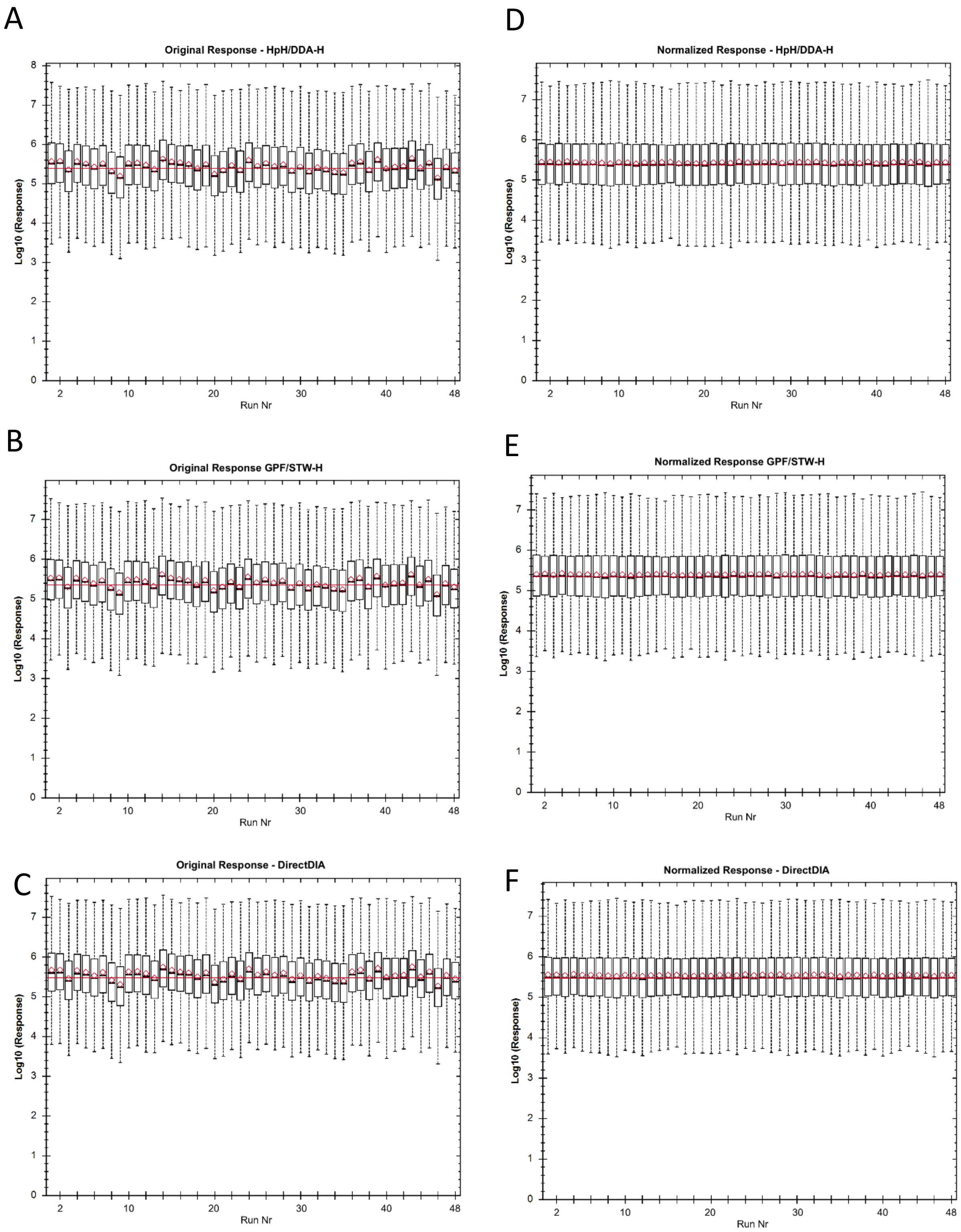

Figure S4. MS/MS level signal normalization of samples analyzed with selected ion-chromatogram libraries and DirectDIA approach. Panels A, B, C –pre-normalization MS2 level detector response of HpH/DDA-H, GPF/STW-H and DirectDIA -analyzed samples, respectively. **Panels D,E,F** – respective post-normalization signal values. Samples were analyzed with staggered windows 24m/z STW method. Individual rectangles denote Q1 (upper line), Median (middle line) and Q3 (lower line) percentile. Mean response value is denoted by red diamonds.

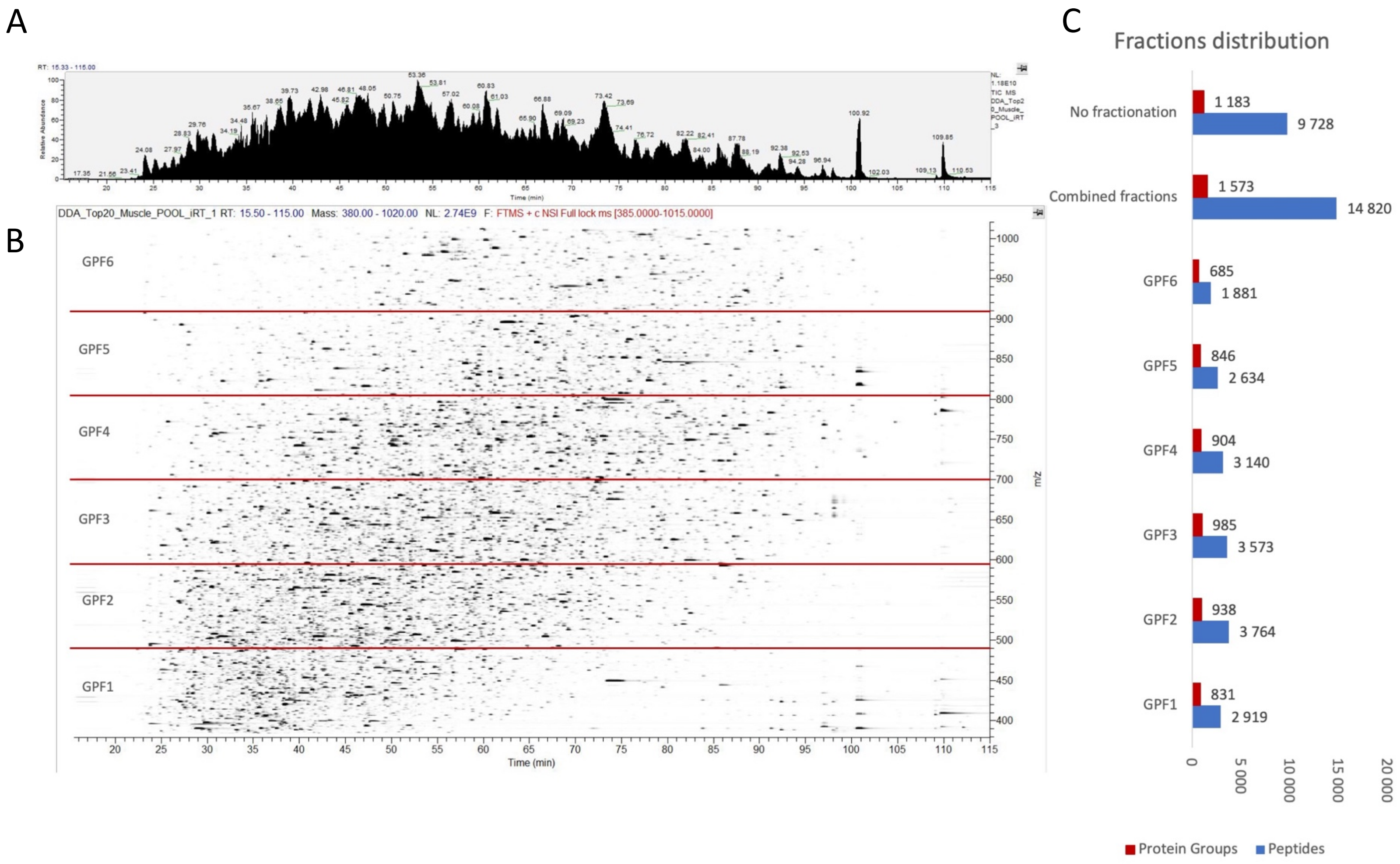

Figure S5. Results of gas-phase fractionation of ions (GPF) for library generation. Panel A – Total ion chromatogram from 750µg of muscle protein digest resolved on 50cm µPAC column. **Panel B** – Corresponding m/z vs time ion heat map. Red lines denote gas-phase mass fraction boundaries. Panel C - unique peptides/protein groups in individual gas-phase mass fractions (4m/z GPF-STW analysis). No fractionation – results from non-fractionated sample (24m/z STW method).

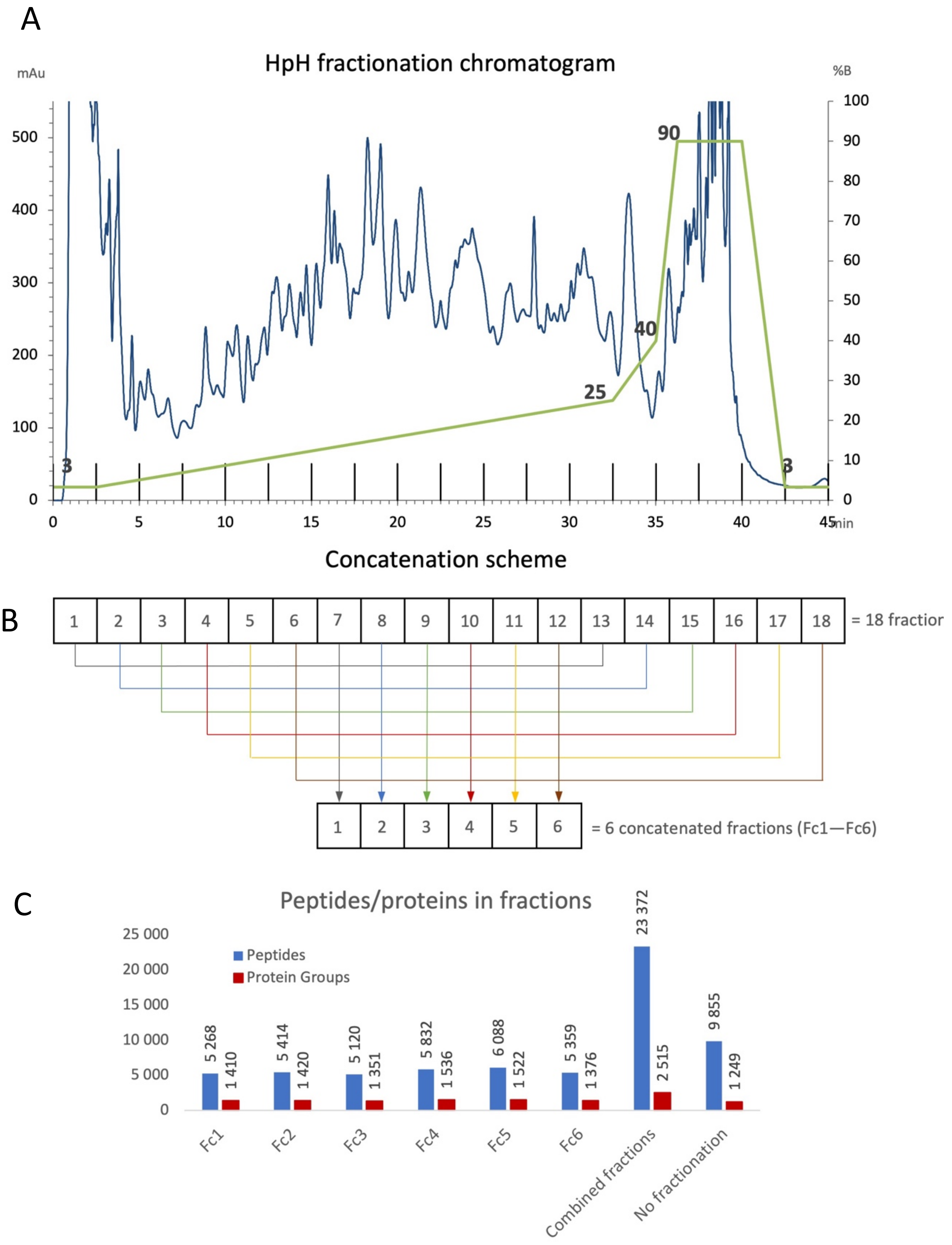

Figure S6. Results of off-line high-pH fractionation (HpH) of peptides for library generation. **Panel A** – 214nm absorbance chromatogram, HpH chromatographic gradient and collected fractions from 100μg of muscular protein digest. **Panel B** – fraction concatenation scheme for the equalization of peptide distribution ion fractions and improvement of orthogonality of HpH and acidic-pH chromatography. **Panel C** – unique peptides/protein groups in individual concatenated fractions (DDA analysis). No fractionation – results from non-fractionated sample (DDA analysis).

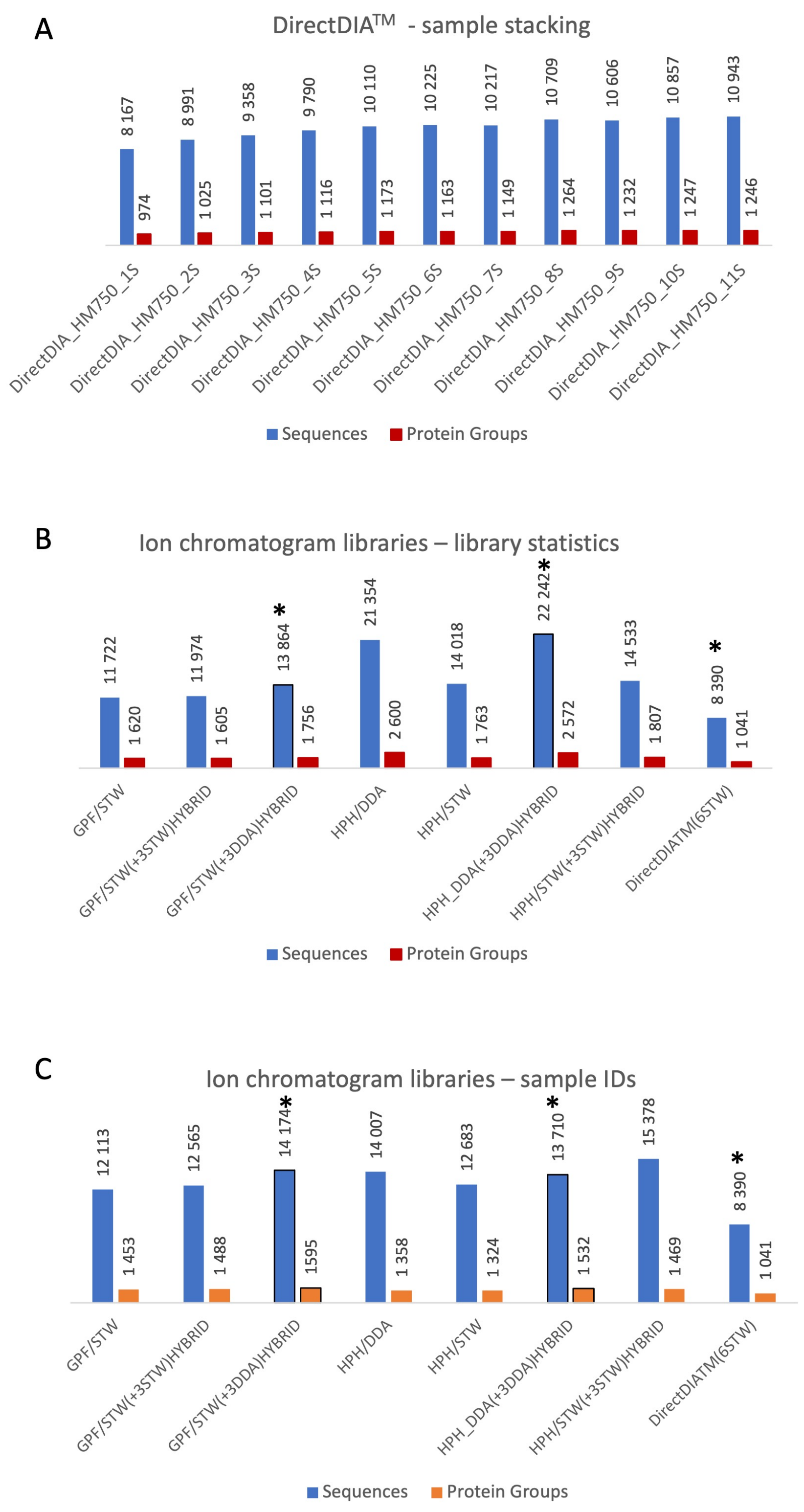

Figure S7. Peptide/protein identifications using different approaches for the generation of ion-chromatogram library. Panel A – The effect of sample runs stacking using multiplicate 24m/z STW sample runs on the number of peptides/protein groups identified with DirectDIA approach. **Panel B** – Basic statistics of ion-chromatogram libraries. Detailed description and results are presented in **Table S5**. **Panel C** – Basic identification results of muscle protein digest (n=3, 24m/z STW method) using appropriate library combination. Detailed description and results are presented **in Table S6**.

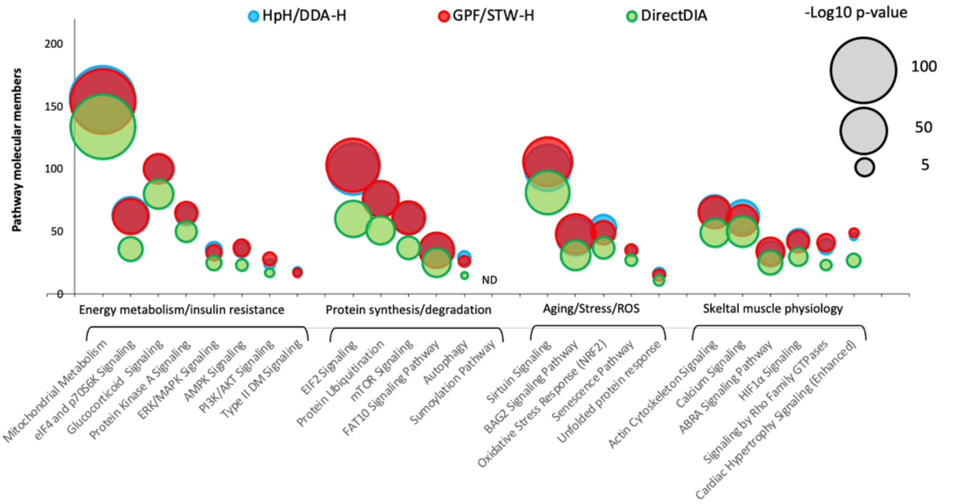

	Significantly enriched pathways:	Mean enrichment -log10(p-value):	Pathway fraction (identified/total):	Mean members/pathway:
HpH/DDA-H	521	7.8	0.29	21.4
GPF/STW-H	558	7.7	0.29	20.9
DirectDIA	472	6.8	0.24	16.4

Figure S8. Pathway enrichment analysis of selected diabetes-related molecular pathways and basic statistics of molecular pathways identified with a given ion-chromatogram library (table insert). Bubble size represents enrichment significance of a given pathway (expressed as –$\log_{10}$(p-value)), whereas Y axis represents the total number of identified molecular members (proteins) of a given pathway.

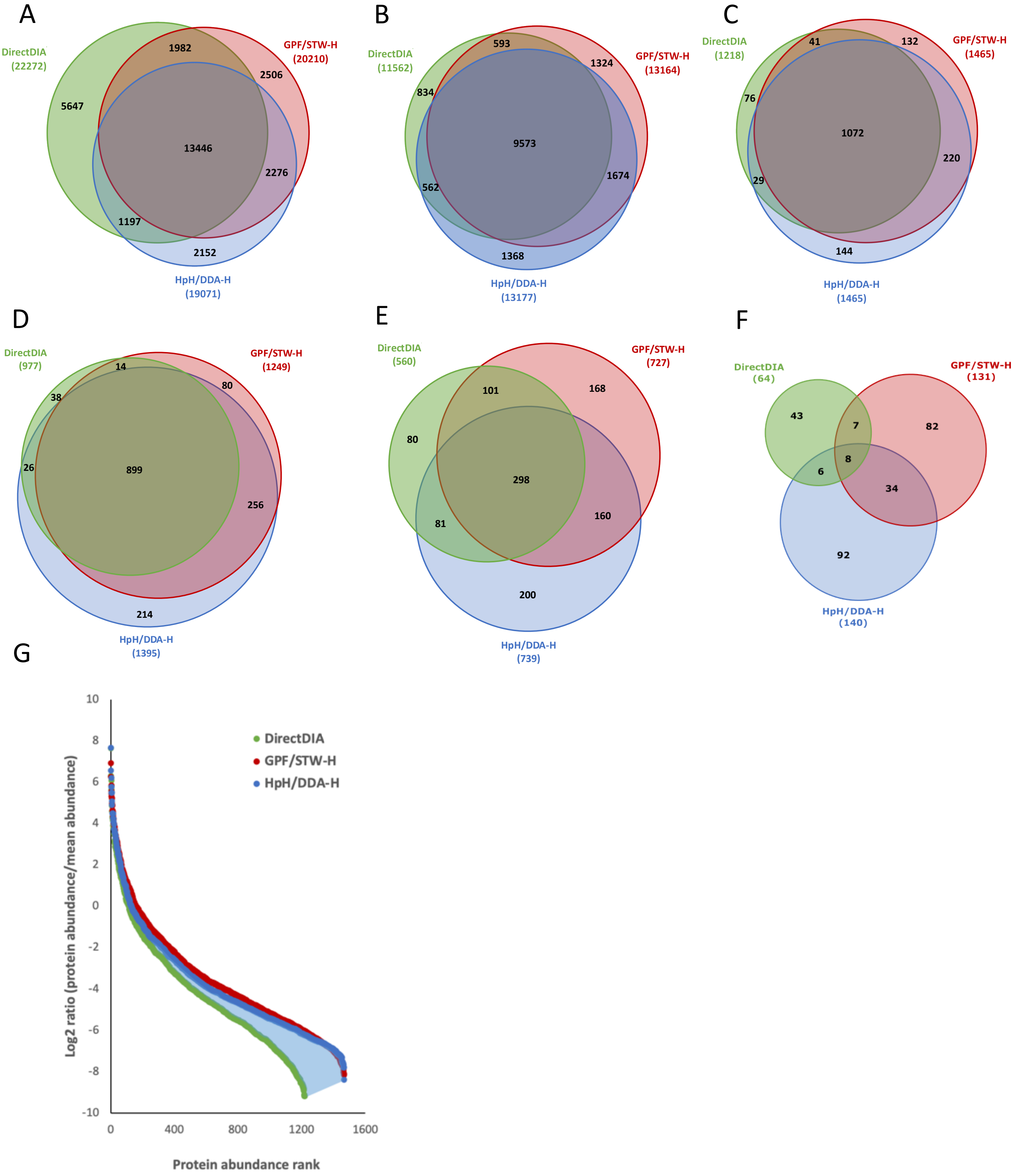

Figure S9. Comparison of the results of IGT vs NGT differential expression analysis at various stages of protein identification and quantitation. Venn diagrams from IGT vs NGT comparison at precursor, peptide and protein level **(Panel A to C)**; Venn diagrams of proteins with ≥2 unique peptides, with FDR-corrected p-value<0.05 (Q-value <0.05) and with ±50% fold change (additively) **(Panel D to E)**; protein rank distribution plot from IGT vs NGT comparison **(Panel F)**

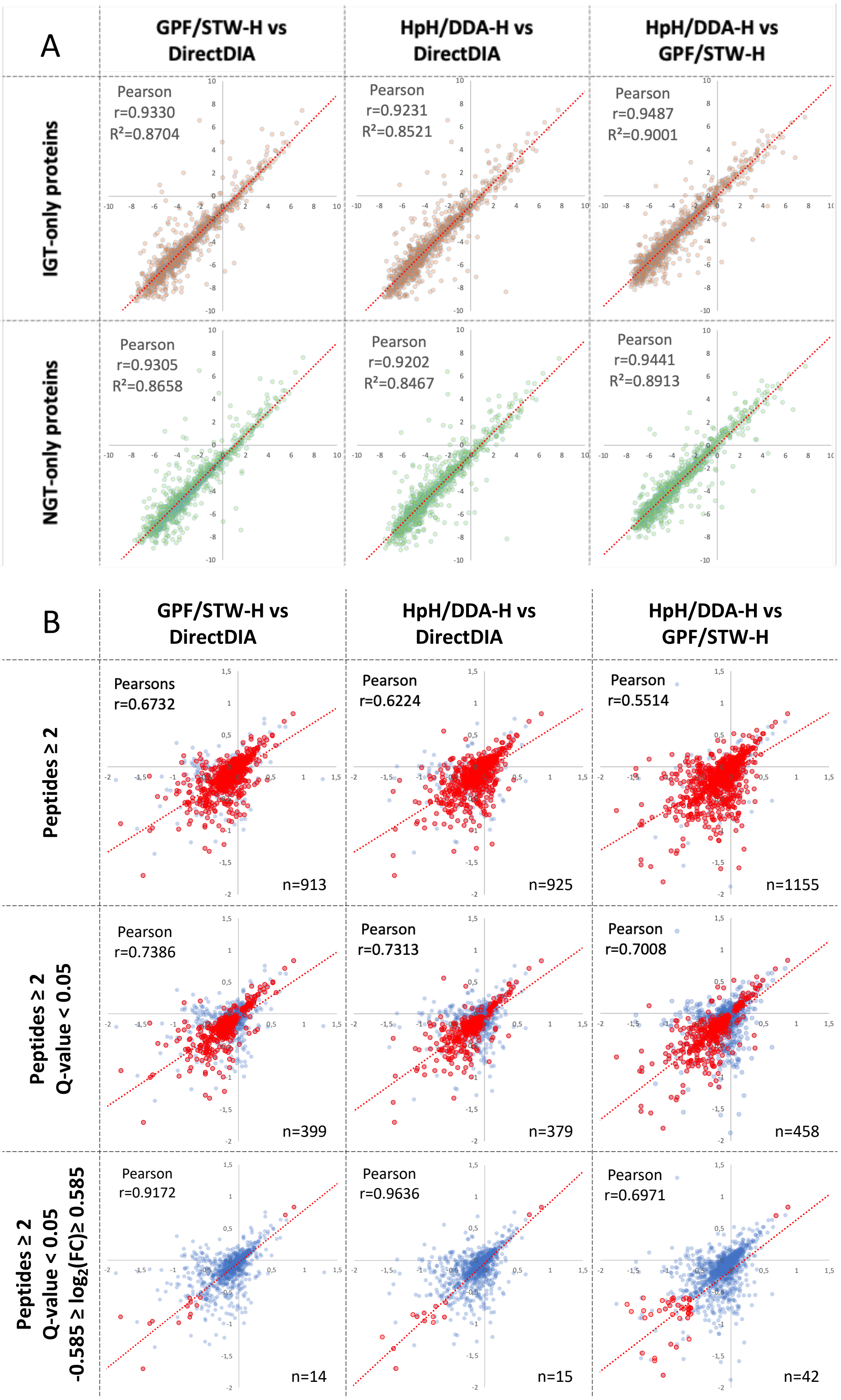

Figure S10. Correlation analysis of protein expression from IGT vs NGT comparison. Correlation plots of respective approaches at the level of IGT- or NGT-only samples **(Panel A);** Correlation of IGT vs NGT differential expression $\log_2$(fold change) of proteins with ≥2 unique peptides, with FDR-corrected p-value<0.05 (Q-value<0.05) and with ±50% difference (additively) **(Panel B)**; 50% fold-change described as -0.585≥$\log_2$(fold change)≥0.585

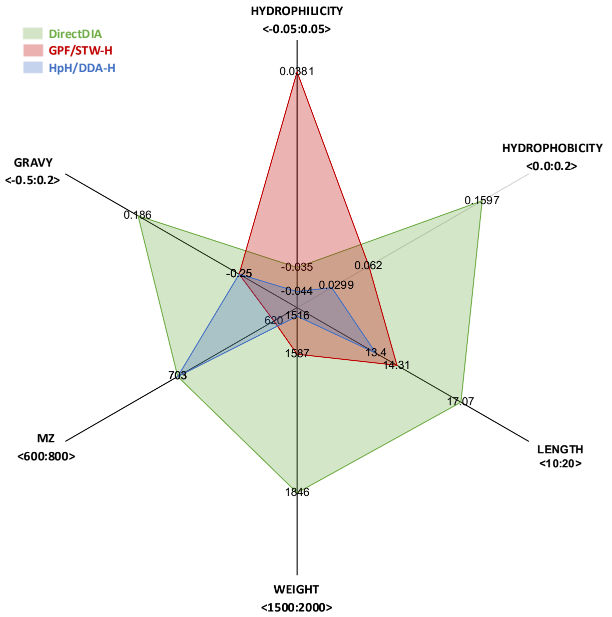

Figure S11. Radar plot of the peptide physicochemical properties from significantly affected proteasomal degradation-related proteins. Proteins from post-exercise IGT/NGT comparison, with ≥2 unique peptides, FDR-corrected p-value<0.05 (Q-value<0.05) and 50% fold-change $-0.585 \geq \log_2(\text{fold change}) \geq 0.585$.
